# Characterizing Sensory Drivers of Acceptance, Purchase Intent, and Emotional Responses to Plant-Based Milk Alternatives in Chilled Sweetened Coffee Among Thai Consumers

**DOI:** 10.3390/foods15152583

**Published:** 2026-07-23

**Authors:** Anh Luu Hoang Nguyen, Siriporn Siralertmukul, Sarisuk Sittiketgorn, Aussama Soontrunnarudrungsri, Suntaree Suwonsichon

**Affiliations:** Kasetsart University Sensory and Consumer Research Center (KUSCR), Department of Product Development, Faculty of Agro-Industry, Kasetsart University, Bangkok 10900, Thailand; anhluuhoang.n@ku.th (A.L.H.N.); sirasi222@gmail.com (S.S.); fagisss@ku.ac.th (S.S.); aussama.s@ku.th (A.S.)

**Keywords:** non-dairy milk alternatives, nut-based milks, coffee pairing, Arabica–Robusta blend, sensory profiles, consumer preference, emotional responses, descriptive analysis

## Abstract

Sensory characteristics play a significant role in driving the market success of plant-based milk alternatives (PBMAs) in coffee applications. This research identified key sensory attributes affecting Thai consumers’ acceptance, purchase intent, and emotional responses toward chilled, sweetened coffee–PBMA formulations prepared with specific commercial products available in Thailand. Ten formulations—combining two coffee bases (100% Arabica or 70:30 w/w Arabica–Robusta blend) with five PBMAs (almond, pistachio, macadamia, white sesame, and riceberry)—were evaluated by nine trained descriptive panelists for 26 attribute intensities and by 100 Thai milk coffee consumers for overall liking, purchase intent, and emotional responses. Results showed that PBMAs primarily determined sensory profiles, consumer acceptance, and elicited emotions, whereas coffee bases exerted marginal influence. Macadamia and pistachio milks exhibited significantly (*p* ≤ 0.05) higher overall acceptance and top-two-box purchase intent than the remaining alternatives, while eliciting strong positive emotional responses. Four attributes, including *nutty*, *beany*, *balanced/blended*, and *fullness*, primarily fostered consumer liking and positive emotions (e.g., *comforted*, *happy*, *healthy*). Conversely, two attributes (*dark brown flavor* and *powdery texture*) served as major sensory barriers, linked to a decline in acceptance and negative emotions. These insights may provide valuable guidance for product developers to optimize specific coffee–PBMA formulations.

## 1. Introduction

The global market for plant-based milk alternatives (PBMAs) is expanding significantly, with projections estimating a rise from USD 2.8 billion in 2022 to USD 7.3 billion by 2032 at a compound annual growth rate (CAGR) of 10.3% from 2023 [1]. This upward trend reflects a growing consumer shift toward functional beverages, particularly among lactose-intolerant and flexitarian populations [2]. PBMAs can be prepared from a diverse range of plant sources, including grains, legumes, nuts, and seeds, among which oat, soy, and almond variants are the most widely consumed. Owing to their exceptional versatility, these PBMAs find applications far beyond conventional milk substitution, extending into sectors such as cheese alternatives, dessert analogs, ice cream, and fermented products. A highly notable and increasingly prevalent application is the integration of PBMAs into the globally expanding coffee culture, where consumers actively seek non-dairy options for lattes, cappuccinos, and specialty coffee beverages. Given that coffee is one of the most widely consumed beverages worldwide, the interaction between coffee and PBMAs has become a focal point for the beverage industry [3]. Therefore, evaluating the sensory characteristics of coffee–PBMA formulations is essential for optimizing product quality, ensuring consumer satisfaction, and supporting the continued innovation and market growth of these functional pairings.

The existing literature on coffee–PBMA formulations has predominantly focused on consumer acceptance, often limited to a narrow selection of plant-based milk types such as oat, soy, almond, and coconut. Consumer acceptance of these hybrid beverages varies widely across different geographical regions, depending on the specific plant source used, as demonstrated by Zakidou et al. [4], who found that Greek consumers accepted cappuccinos prepared with oat and coconut–soy milk but rejected those formulated solely with soy milk. Similarly, Oblitas-Delgado et al. [5] reported varied acceptance among Peruvian consumers for coffee paired with almond, soy, and coconut milk. A comprehensive review by Mekanna et al. [6] further highlighted that regional preferences shape specific flavor profiles; for example, Western European consumers favor less sweet, less beany, and less grainy attributes in plant-based beverages. Despite these insights, cross-cultural sensory data are still evolving, and information on the specific preferences and perceptions of Southeast Asian consumers, notably in Thailand, toward a wider, more varied selection of coffee–PBMA formulations remains limited. Investigating this expanding market not only fills a critical geographical gap but also provides an essential comparative model for cross-cultural sensory science. This aids global beverage developers in understanding how Southeast Asian consumer cohorts perceive diverse PBMAs when paired with coffee.

Beyond acceptance evaluation, understanding the precise relationship between specific sensory attributes and consumer preference is crucial to identifying the drivers of liking for these complex beverage products. While existing research has provided valuable insights into coffee–PBMA formulations, the current literature often utilizes methodological approaches that capture different dimensions of sensory perception. For instance, Gorman et al. [7] utilized the Check-All-That-Apply (CATA) method with Canadian consumers to evaluate coffees formulated with dairy milk and PBMAs, including oat, soy, and almond. Their findings identified sweet, roasted, chocolate, and nutty attributes as positive drivers of liking, whereas beany, earthy, sour, and bitter traits detracted from consumer preference. Chung et al. [8] initially derived a descriptive sensory vocabulary through quantitative descriptive analysis (QDA) but subsequently employed a CATA approach with Taiwanese consumers to investigate hot coffee pairings with cow milk and various PBMAs (oat, soy, almond, and coconut). Their findings indicated that sweetness, smoothness, creaminess, and thickness emerged as the positive drivers of liking, while rancid oil, greasy, astringent, and rice bran attributes functioned as the negative drivers. Although both studies successfully mapped these consumer perceptions, the CATA method inherently characterizes samples based on the presence or absence of sensory attributes from a consumer perspective, rather than quantifying precise attribute intensities evaluated by trained sensory panelists. In research regarding PBMAs as individual beverages, Pramudya et al. [9] demonstrated that combining reference-anchored descriptive analysis by a trained panel with large-scale consumer testing is highly effective for mapping sensory profiles to consumer liking. Adopting a comparable, integrated methodology for complex coffee–PBMA systems—while expanding the investigation to a wider selection of plant-based milk types—would yield profound and actionable insights, enabling a more precise identification of how specific attribute intensities influence consumer preferences. Simultaneously, it is equally important to investigate variations in the coffee base itself, a factor largely overlooked by previous studies that used a single, uniform coffee profile and focused primarily on hot beverage formats. Evaluating the specific interactions between different coffee varieties or blends and diverse plant-based milk bases within cold coffee formulations—a format that dominates consumer preferences across Asian markets, particularly in Thailand [10]—is essential to fully understanding and optimizing these hybrid formulations. Indeed, serving temperature is known to alter sensory dynamics of coffee; lower temperatures suppress the release of key volatiles (e.g., aliphatic ketones, alkylpyrazines, furans, pyridines) and modulate flavor attributes (e.g., bitterness, sweetness, roasted notes, overall intensity) [11,12].

Although sensory attributes determine immediate palatability [13], emotional profiling provides deeper insights into consumer decision-making [14]. In research evaluating PBMAs as individual beverages, Patabandige et al. [15] examined the responses of New Zealander and Singaporean consumers toward oat, soy, and rice milk, demonstrating that dairy-like attributes (e.g., creamy mouthfeel and milk-like flavor) not only drove liking but also evoked positive emotions (e.g., comforted). Conversely, weak or bland profiles triggered negative emotions (e.g., bored or uninspired). While a significant literature gap remains concerning the emotional responses elicited by coffee–PBMA formulations and their correlation with quantified sensory profiles, current research has yet to examine these responses in tandem with the simultaneous interactions of diverse, less-studied PBMAs and varying coffee profiles in cold sweetened beverage formats.

To address the holistic gap, this study aimed to characterize the sensory profile, consumer acceptance, and emotional responses elicited by chilled sweetened coffee–PBMA systems. In doing so, the present work focuses on two core questions: (1) how the integration of diverse PBMAs, including less-studied types, with different coffee bases affects the descriptive sensory profiles of the matrices, and (2) how these variations subsequently drive Thai consumers’ liking, purchase intent, and emotional engagement. To achieve this, reference-anchored descriptive analysis utilizing well-defined sensory descriptors was employed to evaluate ten distinct formulations combining two coffee bases—100% ground Arabica and a 70:30 (w/w) Arabica–Robusta blend—with five selected PBMAs. These alternatives comprised globally established nut milks (almond, pistachio, macadamia) alongside local plant-based milks of cultural and economic importance: white sesame (a lipid- and nutrient-rich seed system) [16] and riceberry (a starch-rich carbohydrate system offering antioxidant health benefits from anthocyanins) [17]. Concurrently, consumer acceptance, purchase intent, and emotional profiling were assessed among Thai consumers. By modeling the interrelationships across quantified sensory intensities, liking scores, purchase intent, and emotional data, this research identifies the key drivers of consumer preference and emotional engagement, providing actionable insights for product optimization in the rapidly expanding Southeast Asian market. Ultimately, evaluating these distinct regional raw materials provides the international scientific community with a valuable benchmark for how non-traditional plant-based milk matrices perform in complex coffee systems.

## 2. Materials and Methods

### 2.1. Coffee and PBMAs

Two medium-roast coffees and five PBMAs were procured. The coffee varieties included 100% ground Arabica (Coffee Roasting Co., Ltd., Nakhon Ratchasima, Thailand) and 100% ground Robusta (Wiengsa origin, Doi Coffee, Nan, Thailand). A coffee blend consisting of Arabica and Robusta at a 70:30 (w/w) ratio was then prepared for experimental use. The PBMAs included almond and macadamia milk (137 Degrees^®^, Simple Foods Co., Ltd., Pathum Thani, Thailand); pistachio milk (Wholly Nuts^®^, Simple Foods Co., Ltd., Pathum Thani, Thailand); white sesame milk (Sesamilk, Sasamilk Foods Co., Ltd., Bangkok, Thailand); and riceberry milk (4Care, 4CARE Co., Ltd., Bangkok, Thailand). These PBMAs were unsweetened, except for the riceberry milk, which was sweetened with 4% sugar. All coffee and PBMA materials were commercially available in the Thai market and were purchased from local retail outlets. Detailed information regarding the ingredients and nutritional composition of each PBMA, as declared on their respective commercial packaging, is provided in Table A1 (Appendix A).

### 2.2. Preparation of Coffee–PBMA Samples

Coffee samples were prepared using either 100% ground Arabica or an Arabica–Robusta blend (70:30, w/w). Each coffee base was combined with five distinct PBMAs (almond, pistachio, macadamia, white sesame, and riceberry), yielding a total of ten coffee–PBMA samples for testing.

Espresso was brewed using a LELIT espresso machine (model PL41QEP, Lelit S.r.l., Brescia, Italy). For each sample, 18 g of ground coffee was placed into the machine’s portafilter and extracted with 36 mL of water, yielding 30 mL of espresso. Subsequently, 12 g of pure refined sugar (Mitr Phol Sugar Corp., Bangkok, Thailand) and 175 mL of a specific PBMA were added, and the mixture was stirred until the sugar dissolved completely. The standardized sugar level (12 g) was applied across all formulations to reflect common Thai consumer and food service practices, where a consistent recipe is typically maintained regardless of the variant or inherent sweetness of the commercial PBMAs selected. Additionally, preliminary benchtop tastings indicated that reducing the added sugar specifically for the coffee–riceberry milk formulation resulted in an unappealingly thin body. This procedure was repeated for each sample until a sufficient volume was obtained for sensory evaluation. The resulting espresso-to-PBMA ratio was 1:5.8 (v/v), which is comparable to typical latte formulations that generally feature espresso-to-milk ratios ranging from 1:5 to 1:6 (v/v) [18]. Prior to sensory evaluation, the prepared samples were stored in a refrigerator (4–5 °C) for approximately 1 h and served chilled. Although these standard proportions are traditionally established for hot coffee systems, maintaining a 1:5.8 ratio remains highly relevant for chilled formulations. At lower serving temperatures, volatile aroma release is suppressed and flavor attributes, such as bitterness and sweetness, are modulated [11,12]. Consequently, this specific ratio provides a sufficient volume of the PBMA matrix to effectively balance the altered sensory profile of the coffee solids while preserving the desired body and mouthfeel in the chilled state.

### 2.3. Sensory Evaluation

The ten coffee–PBMA samples were evaluated using descriptive analysis to characterize their sensory profiles, and an acceptance test to assess consumer liking, purchase intent, and emotional responses. All testing was conducted at the sensory evaluation facilities of the Department of Product Development, Kasetsart University, Bangkok, Thailand. The testing room was physically separated from the sample preparation area. The evaluation environment featured appropriate lighting, was air-conditioned (25 °C), and remained free from extraneous odors. The involvement of human subjects in this research was approved by the Kasetsart University Research Ethics Committee (approval number COE67/081). Written informed consent was obtained from all participants prior to testing.

#### 2.3.1. Descriptive Analysis

Descriptive analysis, based on a profile method adapted from Keane [19], was performed by nine trained panelists (all females, age range 40–58 years) affiliated with the Kasetsart University Sensory and Consumer Research (KUSCR) Center. The panelists had completed 120 h of descriptive analysis training and possessed at least 2000 h of testing experience with a variety of food products and beverages, including brewed coffee and PBMAs. These panelists were selected and utilized as analytical instruments based on their sensory acuity and performance, rather than as consumer representatives; hence, demographic diversity, such as gender and age, was not required. The number of trained panelists used in this research corresponded to the ranges reported in the literature for descriptive analysis; Heymann et al. [20] suggested 8–12 panelists, while Maximo-Gacula and Rutenback [21] and Drake [22] recommended 6–14 panelists.

Orientation was conducted across four 3 h sessions to familiarize the panelists with the samples and to facilitate sensory lexicon development. During these sessions, the panelists individually evaluated the coffee–PBMA samples and identified a set of terms to describe their flavor and texture characteristics. An initial list of terms from previous studies on brewed coffee [23,24] and PBMAs [7,8,9] was provided to assist in term identification. However, the panelists were permitted to include additional terms at their discretion, regardless of whether they were on the initial list. The entire panel then discussed the proposed terms, compiled a final list of attributes for testing, and reached a consensus on attribute definitions, reference standards, and intensities.

Subsequently, two 3 h training sessions were conducted during which the panelists practiced scoring each attribute on a 15 cm line scale, where 0 represented “none” and 15 represented “extremely high”. Panel performance was monitored to ensure that the panelists’ scores were aligned and the inter-panelist variation was minimized.

Thereafter, product testing was performed across four 3 h sessions over 2 days (one morning and one afternoon session per day). For these tests, two replications were evaluated for each of the ten coffee–PBMA samples, resulting in a total of 20 evaluations per panelist, with a maximum of 5 samples presented in each session. For each evaluation, panelists received 30 mL of the sample at 4–5 °C in an odorless 2-oz. plastic cup labeled with a three-digit random number, and the serving order was randomized within each replication. After tasting each sample, the panelists rated the intensity of all attributes on the 15 cm line scale, with references provided during evaluations to anchor the values on the scales. To mitigate sensory fatigue, caffeine accumulation, and carry-over effects, a mandatory 15 min break was enforced after each sample evaluation, during which panelists cleansed their palates using reverse-osmosis deionized water, sliced apples, and unsalted crackers (Jacob’s Original Cream cracker, Kraft Foods Malaysia, Petaling Jaya, Malaysia). This protocol provided a generous buffer of 36 min per sample slot, ensuring sufficient sensory recovery before the next evaluation.

#### 2.3.2. Acceptance Test

One hundred Thai consumers (72 females and 28 males, aged 18–65 years) participated in the study. Participants were screened based on the criteria that they consumed milk coffee at least three times a week, had no history of food allergies, and had no aversion to PBMAs. Among the 100 participants, 7 consumed milk coffee more than once daily, 35 once daily, and 58 three to five times per week. Bhumiratana et al. [25] categorized consumers who drank coffee at least once daily, 3–5 times/week, and 1–2 times/week as heavy, medium, and light users, respectively. Consequently, the participant cohort comprised 42 heavy users and 58 medium users of milk coffee. To account for familiarity with plant-based beverages, participants were asked about their previous experience with PBMAs; 91% of the participants reported prior consumption of plant-based milk, including almond, pistachio, riceberry, soy, oat, coconut, corn, walnut, macadamia, and sesame milk. The remaining 9% who had no prior experience with PBMAs were classified as active milk coffee consumers (3 heavy and 6 medium users), representing a potential consumer segment open to PBMA–coffee beverages.

Each consumer evaluated all ten coffee–PBMA samples across two sessions, with five samples evaluated per session. Evaluations were separated by a 5 min break between samples and a 20 min break between sessions. Samples (30 mL) at 4–5 °C were monadically served in odorless plastic cups (2 oz.) labeled with three-digit random codes, following a balanced and randomized order based on a Williams Latin square design. After tasting each sample, participants were instructed to rate their overall liking on a 9-point hedonic scale (1 = dislike extremely, 5 = neither like nor dislike, 9 = like extremely) and their purchase intent on a 5-point category scale (1 = definitely would not purchase, 3 = might or might not purchase, 5 = definitely would purchase). Participants also completed a check-all-that-apply (CATA) question to describe the emotional responses evoked by each coffee–PBMA sample. The CATA question included 25 emotion terms (Table 1) derived from the study by Pinsuwan et al. [23] on the emotions experienced by Thai consumers while drinking brewed black coffee, as well as from the EsSense Profile^TM^ (ESP) [26], the WellSense Profile^TM^ [27], and the Coffee-Drinking Experience (COE) Profile [25]. The emotion terms were presented in a random order across consumers to minimize position bias, in accordance with previous studies [23,28,29]. Unsalted crackers (Jacob’s Original Cream cracker, Kraft Foods Malaysia, Petaling Jaya, Malaysia) and reverse-osmosis deionized water were provided for palate cleansing between samples.

### 2.4. Data Analysis

Analysis of variance (ANOVA) was performed to determine significant differences among ten coffee–PBMA samples based on attribute intensities and overall liking scores at a 95% confidence level (*p* ≤ 0.05). For the attribute intensity data, the effects of sample, panelist, replication, and their two-way interactions were included in the ANOVA model, while the three-way interaction (sample × panelist × replication) was used as the error term. For the overall liking data, a Linear Mixed Model (LMM) was implemented to account for the repeated-measures structure of the consumer test, with sample as a fixed effect and consumer as a random effect. Mean comparisons for significant differences among the samples in both datasets were subsequently determined using Duncan’s Multiple Range Test (DMRT). Principal component analysis (PCA) with varimax rotation was then conducted to visualize the underlying relationships between the sensory attributes and the samples. To justify the use of this orthogonal rotation against potential intercorrelations among sensory modalities, oblique rotations (Oblimin and Promax) were additionally examined. The correlation coefficients between the factor scores of Dimension 1 (D1) and Dimension 2 (D2) were near-zero (r=0.0086 for Oblimin and r=−0.0391 for Promax), confirming that the extracted dimensions were independent and that the Varimax approach was highly appropriate for this dataset. Hierarchical cluster analysis (HCA) was performed using Ward’s minimum variance method to categorize the samples into groups with similar sensory characteristics. Cochran’s Q test was used to determine significant differences among the samples based on the frequency counts of each emotion term at a 95% confidence level (*p* ≤ 0.05). When significant differences were detected, Sheskin’s critical difference method was applied for multiple pairwise comparisons. Correspondence analysis (CA) was then performed to visualize the relationships between the emotion terms and the samples. Additionally, purchase intent data were analyzed using the same statistical approach (Cochran’s Q and Sheskin’s tests) after transforming the 5-point scale responses into a binary format (scores of 1–3 coded as 0; 4–5 coded as 1). Partial least squares regression (PLSR) analysis was also conducted to identify the sensory drivers of liking, purchase intent, and emotional responses, using sensory attribute intensities as X-variables and overall liking scores, top-two-box purchase intent (%), and emotional responses (proportion of frequency counts) as Y-variables. Non-significant sensory attributes (based on ANOVA) and emotion terms (based on Cochran’s Q test) were removed from the dataset prior to PCA, HCA, CA, and PLSR analysis. Statistical analyses for ANOVA and DMRT were performed using IBM SPSS Statistics version 28.0 (Thaisoftup Co., Ltd., Bangkok, Thailand), while PCA, HCA, Cochran’s Q test, CA, and PLSR analysis were executed using XLSTAT statistical software version 2025.1.3, build 1431 (Addinsoft, Paris, France).

## 3. Results and Discussion

### 3.1. Sensory Characteristics

Table 2 presents the sensory lexicon of 26 terms, along with their definitions, reference standards, and intensities, used to evaluate the flavor and texture characteristics of coffee–PBMA samples in this study. Seventeen terms were adapted from previous studies on brewed coffee [23,24] and PBMAs [7,8,9] with certain sensory references modified as necessary to address limited availability and to better align with the products under evaluation. The remaining nine terms—specifically *brown*, *cooked*, *dark brown*, *wax*, *sesame*, *gel-like*, *body*, *powdery*, and *mouth coating*—were newly developed through panel discussion and consensus during the lexicon development process. A well-defined sensory lexicon with appropriate reference standards enables accurate, precise, and consistent communication among sensory panelists, thereby enhancing the reliability of the experimental results [30]. A clear demonstration of this principle was provided during the calibration of the newly developed attributes *brown* and *dark brown*. Although both fall within the thermal aromatic spectrum, potential perception overlap was effectively resolved by providing distinct definitions and physical anchors. The brown attribute was defined and anchored to represent moderate thermal progression (using pinto beans), whereas dark brown captured an intense flavor of highly heated foods that are almost burnt (using a diluted chocolate syrup solution). Presenting these specific reference standards side-by-side enabled the panel to align their criteria, focusing on these distinct stages of thermal intensity until a strict group consensus was successfully achieved prior to actual product testing.

Mean intensity scores for the sensory attributes of the coffee–PBMA samples prepared from either 100% ground Arabica or the 70:30 (w/w) Arabica–Robusta blend, each combined with almond, pistachio, macadamia, white sesame, or riceberry milk, are presented in Table 3. ANOVA and DMRT results indicated that 24 of the 26 attributes differed significantly (*p* ≤ 0.05) among the samples. Substantial differences were observed primarily for *nutty*, *beany*, *dark brown*, *caramel*, *grain*, *sesame*, *balanced/blended*, *body*, and *fat feel*. Notably, the statistical significance (*p ≤* 0.05) observed for *dark brown*, contrasted with the non-significant effect found for *brown*, empirically validates that the trained panel successfully discriminated between these two attributes without perception overlap.

Regarding panel reliability, a significant replication effect was found in 12 attributes, suggesting slight shifts in scale usage between replications. However, the panelist × replication interaction was significant in only 4 attributes, demonstrating that individual panelists maintained high repeatability and consistent scoring behavior across the two replications. Consequently, although some numerical differences among the sensory attributes appeared relatively small on the 15 cm scale, their statistical significance (*p* ≤ 0.05) remains highly valid. Due to the high acuity of this rigorously trained panel, their consistent scoring behavior, and the use of standardized references, these subtle shifts reflect genuine variations in the product matrices rather than panel noise. From a product development perspective, capturing these minor sensory changes is critical for precise formulation optimization, as even subtle alterations in attributes could influence overall sensory harmony and potentially modulate consumer acceptance towards the coffee–PBMA samples.

**Figure 1:**
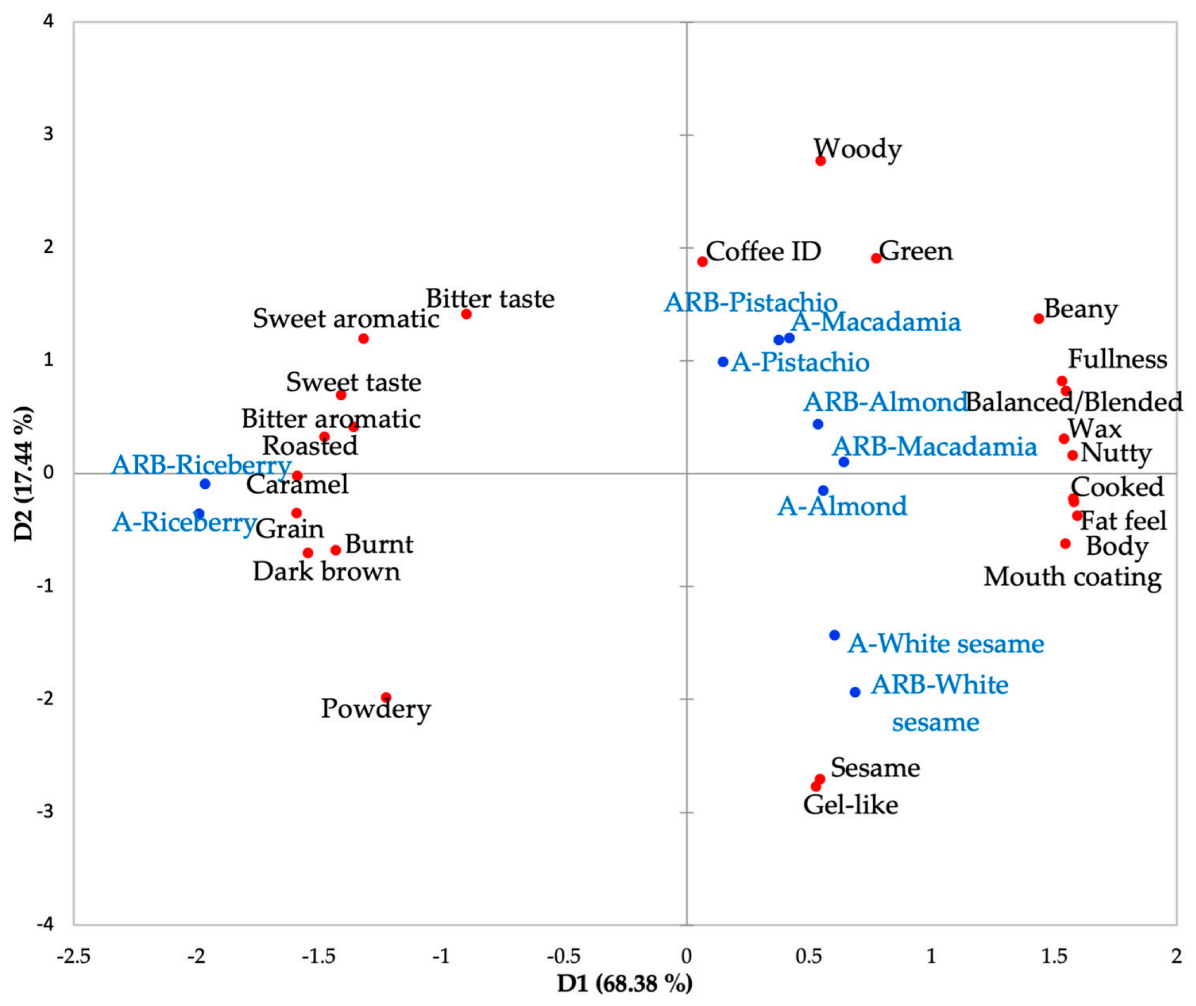
PCA biplot illustrating the relationships between sensory attributes and chilled sweetened coffee–PBMA samples prepared from 100% ground Arabica (A) or the 70:30 (w/w) Arabica–Robusta blend (ARB), each combined with almond, pistachio, macadamia, white sesame, or riceberry milk.

PCA results indicated that the significant sensory attributes were grouped into two principal components (PCs), accounting for 85.82% of the total variance (68.38% and 17.44% for PC1 and PC2, respectively), as illustrated in Figure 1. On PC1, attributes with absolute loadings > 0.6 [23] included *nutty*, *cooked*, *beany*, *wax*, *balanced/blended*, *fullness*, *body*, *fat feel*, and *mouth coating* in the positive dimension, and *roasted*, *sweet aromatic*, *burnt*, *bitter aromatic*, *dark brown*, *caramel*, *grain*, *sweet taste*, *bitter taste*, and *powdery* in the negative dimension. On PC2, *woody* loaded heavily in the positive dimension, whereas *sesame* and *gel-like* loaded heavily in the negative dimension. Based on HCA, the coffee–PBMA samples were classified into three distinct clusters. Cluster 1 comprised both the 100% Arabica and Arabica–Robusta blend samples prepared with riceberry milk. Cluster 2 consisted of the Arabica and Arabica–Robusta blend samples combined with almond, pistachio, or macadamia milk, whereas those prepared with white sesame milk formed Cluster 3.

Attributes associated with PC1 mainly differentiated the coffee–PBMA samples in Cluster 1 from those in Clusters 2 and 3. As shown in Table 3, both the 100% Arabica and Arabica–Robusta blend coffees prepared with riceberry milk (Cluster 1) exhibited higher intensities of coffee-related attributes (*roasted*, *burnt*, *bitter aromatic*, *dark brown*, and *caramel*) and *sweet taste*, alongside a more pronounced *powdery* mouthfeel (*p* ≤ 0.05). Conversely, these samples showed lower intensities of *nutty*, *cooked*, *beany*, *balanced/blended*, *fullness*, *body*, *fat feel*, *and mouth coating* compared to those prepared with almond, pistachio, macadamia (Cluster 2), or white sesame (Cluster 3) milk (*p* ≤ 0.05). The prominence of core coffee-derived notes in the Cluster 1 samples is likely due to the relatively mild, cereal-like flavor profile of riceberry milk [31], which minimizes masking effects. The heightened sweetness in these samples is directly attributed to the 4% added sugar inherently present in the commercial riceberry milk used (Table A1, Appendix A), despite the standardized amount of sugar added during formulation preparation. Regarding mouthfeel, the powdery sensation in the riceberry milk coffees is hypothesized to stem from the low swelling power of its starch granules [32] and from amylose retrogradation, which potentially leads to the formation of starch aggregates [33]. This supposition is further corroborated by Magwere et al. [34], who reported that rice milk has a significantly larger average particle size (4.19 μm) than almond, coconut, oat, and soy milk, suggesting a plausible link to the increased powdery sensation observed in this study. Furthermore, the coffees with riceberry milk were characterized by a distinct grain note, which may have been driven by volatiles such as hexanal, nonanal, 2-pentylfuran, and 2-acetyl-1-pyrroline [35]. In contrast, nut- and seed-based milks introduce inherent nutty, beany, roasted, and woody notes and a higher fat content [36,37] (Table A1, Appendix A). This increased lipid fraction likely enhances the fat feel and mouth coating, thereby contributing to a more balanced and integrated flavor profile [31].

Attributes associated with both PC1 and PC2 explained the differences between the coffee–PBMA samples in Clusters 2 and 3. Both the 100% Arabica and Arabica–Robusta blend coffees prepared with almond, pistachio, or macadamia milk (Cluster 2) were significantly (*p* ≤ 0.05) distinguished from those prepared with sesame milk (Cluster 3) by a higher *beany* intensity and stronger tendencies toward *sweet aromatic*, *woody*, *caramel*, and *sweet taste* (Table 3). These profiles are likely mediated by the volatile composition of nut-based milks, which provide beany- and caramel-linked volatiles, such as aldehydes, pyrazines, furans, phenols, and alcohols [38]. Conversely, the samples in Cluster 3 were uniquely characterized by a prominent *sesame* note and exhibited significantly (*p* ≤ 0.05) higher intensities in textural attributes, specifically *gel-like*, *body*, *powdery*, *fat feel*, and *mouth coating*, compared to their nut-milk counterparts in Cluster 2. This relationship is likely driven by the high fat content of sesame milk (Table A1, Appendix A), which subsequently enhances the fat feel and mouth coating [16]. Furthermore, sesame proteins possess high ionic hydration and low hydrophobicity, allowing them to effectively retain water and oil, potentially forming a dense, cohesive protein–lipid matrix that intensifies gel-like, body, and powdery sensations [39]. Although the samples within Cluster 2 shared some common characteristics, variations in certain attributes were observed. Specifically, Arabica coffee with pistachio milk was rated higher for *beany*, while that with almond milk was rated higher for *gel-like* than other samples within the cluster (*p* ≤ 0.05). Arabica and Arabica–Robusta blend coffees with macadamia milk tended to be rated higher for *nutty* and *sweet taste* than the others (*p* ≤ 0.05). It is worth noting that the observed sensory differences among the coffee–PBMA formulations may also reflect the inherent nutritional composition differences among the commercial PBMAs used (Table A1, Appendix A), rather than reflecting the effects of the plant source alone. 

In contrast to the dominant role of the PBMA type, the influence of the coffee type (100% Arabica vs. 70:30 w/w Arabica–Robusta blend) was marginal and inconsistent, often manifesting only in specific attributes and varying according to the PBMA base used. For example, the effect of the coffee type on *bitter taste* was significant (*p* ≤ 0.05) only when pistachio milk was used, with the Arabica–Robusta blend coffee perceived as more *bitter* than the 100% Arabica coffee (Table 3). A similar pattern was observed for *coffee ID*, where the 100% Arabica coffee prepared with macadamia milk exhibited a stronger coffee flavor than the Arabica–Robusta blend (*p* ≤ 0.05), whereas no significant differences (*p* > 0.05) were observed for the other milk types. Furthermore, the *beany* flavor was more intense in the 100% Arabica than in the Arabica–Robusta blend when prepared with pistachio or riceberry milk (*p* ≤ 0.05). These inconsistent findings suggest that the sensory differences between the tested 100% Arabica coffee and the Arabica–Robusta blend appeared to be mitigated by the complex food matrix of the PBMAs, likely due to flavor–protein binding and the masking effect of lipids [40,41].

### 3.2. Consumer Liking, Purchase Intent, and Emotions

Overall liking scores for both the 100% Arabica and Arabica–Robusta blend coffees prepared with macadamia or pistachio milk exceeded 6 (like slightly) on the 9-point hedonic scale. Conversely, the scores for those prepared with almond, white sesame, or riceberry milk remained below 6 (Table 4). While previous studies [4,5,7,8] have consistently identified oat and soy milk as the preferred dairy substitutes for coffee-based applications due to their dairy-like creaminess and mouthfeel, our findings demonstrate that macadamia and pistachio milk emerge as promising alternatives alongside existing commercial plant-based options. Notably, to the best of our knowledge, this study is the first to investigate consumer responses to riceberry and white sesame milk in coffee applications. Although these novel bases—along with almond milk—received lower acceptance scores, documenting their performance provides a foundational sensory baseline for the development of regionally diverse PBMAs.

Regarding the influence of coffee type, Arabica coffee with macadamia milk achieved the highest overall liking score, significantly (*p* ≤ 0.05) outperforming its Arabica–Robusta blend counterpart. In contrast, no significant differences in liking were observed between the two coffee types when prepared with pistachio, almond, white sesame, or riceberry milk. The significantly higher preference for Arabica coffee with macadamia milk suggests a unique sensory synergy; its delicate profile appears to better complement the refined acidity and flavor of 100% Arabica. This was further supported by descriptive analysis, which revealed that the Arabica pairing achieved a significantly (*p* ≤ 0.05) more pronounced *coffee* flavor, alongside numerically higher mean scores for *balanced/blended* and *fullness*, than its Arabica–Robusta counterpart (Table 3).

The results for purchase intent followed a trend similar to that observed for overall liking (Table 4). The top-two-box purchase intent values for both the 100% Arabica and Arabica–Robusta blend coffees prepared with macadamia or pistachio milk were in the higher range (>60%), in contrast to the lower range (28–42%) observed for the coffees prepared with almond, white sesame, or riceberry milk. Notably, the 100% Arabica coffee with macadamia milk achieved the highest top-two-box purchase intent (84%), followed by the Arabica–Robusta blend coffee with pistachio milk (69%). While these two samples did not differ significantly from each other, both exhibited significantly (*p* ≤ 0.05) higher top-two-box purchase intent compared to all other samples. Purchase intention is widely used in consumer research to indicate the likelihood of product purchase [42]. Although no widely accepted criterion has been established, the top-two-box purchase intent values of approximately 60% or higher have been reported in consumer studies (e.g., Mora et al. [43]) as reflecting a relatively high and positive purchase intention when compared across samples. The high top-two-box purchase intent for macadamia and pistachio milk suggests that these nut-based milks possess a premium appeal that aligns with coffee consumers’ preferences, potentially positioning them as competitive alternatives to widely accepted dairy substitutes such as oat and soy milk.

**Figure 2:**
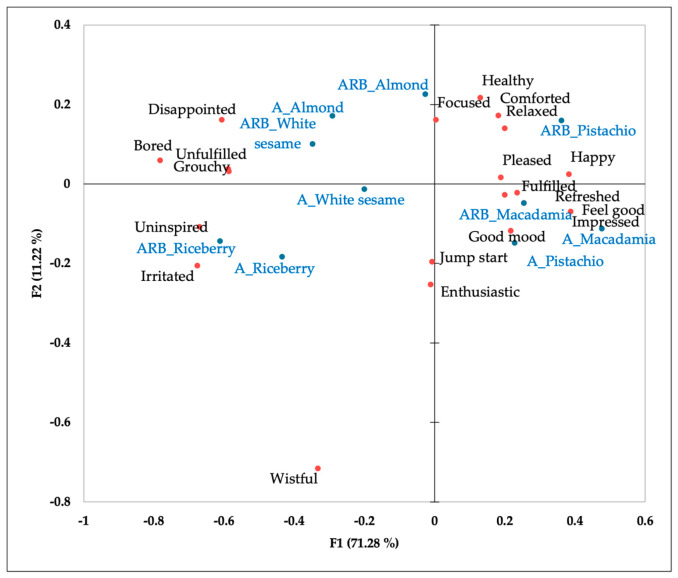
Correspondence analysis (CA) map showing the positioning of chilled sweetened coffee–PBMA samples prepared from 100% ground Arabica (A) or the 70:30 (w/w) Arabica–Robusta blend (ARB), each combined with almond, pistachio, macadamia, white sesame, or riceberry milk in the emotion space. PBMA = plant-based milk alternative.

The results from Cochran’s Q test indicated significant (*p* ≤ 0.05) differences among the ten coffee–PBMA samples for 20 out of 25 emotion terms (Table A2, Appendix A). The emotion terms that did not significantly (*p* > 0.05) discriminate among the samples were *active*, *alert*, *awake*, *energetic*, and *vigorous*. Although Cochran’s Q test showed a significant overall difference among the samples for *enthusiastic* and *wistful*, the subsequent Sheskin post hoc comparisons failed to identify significant pairwise differences. A symmetrical correspondence analysis (CA) map (Figure 2) illustrates the positioning of each coffee–PBMA sample in the emotion space, accounting for 82.5% of the total variance. The Chi-square test of independence confirmed a highly significant association between samples and emotion terms (*p* < 0.0001). Furthermore, based on the average inertia criterion for a 10-sample by 20-emotion term table, the baseline threshold for an axis to be considered meaningful is 1/min (10–1, 20–1) = 1/9 = 11.11%. Both F1 (71.28%) and F2 (11.22%) exceeded this benchmark, confirming that both dimensions capture non-random structures and are statistically meaningful for interpretation.

Similar to the results for overall liking and purchase intent, the PBMA type exerted a greater influence on the emotional profile than the coffee type. The four most preferred samples, comprising the 100% Arabica and Arabica–Robusta blend coffees prepared with macadamia or pistachio milk, were predominantly characterized by positive emotions, with terms including *comforted*, *feel good*, *fulfilled*, *good mood*, *happy*, *healthy*, *impressed*, *jump start*, *pleased*, *refreshed*, and *relaxed* being elicited more frequently than for the other samples (Table A2, Appendix A). Conversely, the samples in the neutral liking range (scores of 5.0–5.6), comprising the 100% Arabica and Arabica–Robusta blend coffees prepared with almond, sesame, or riceberry milk, were associated with negative emotion footprints, characterized by relatively higher proportions of *bored*, *disappointed*, *grouchy*, *irritated*, *unfulfilled*, and *uninspired* (Table A2, Appendix A). These relative associations and positions in the emotion space are visually mapped in Figure 2. Overall, the emotional responses elicited were consistent with the liking scores: more preferred samples evoked more positive feelings, whereas neutral or disliked samples triggered more negative emotions. In some cases, the emotional profiling data offered more detailed insights. For instance, the 100% Arabica coffee with almond milk was perceived as *healthy* (*p* ≤ 0.05) more frequently than the 100% Arabica and Arabica–Robusta blend coffees with riceberry milk (Table A2, Appendix A), despite all three samples showing no significant differences in overall liking (*p* > 0.05) (Table 4).

While PBMAs are generally perceived to evoke neutral or negative emotions compared to the positive emotions elicited by dairy milk [15], our findings demonstrate that the coffees prepared with macadamia or pistachio milk can successfully elicit a wide range of positive emotions. This indicates that specific nut-based milks perform better in a coffee context than others. Furthermore, although previous research indicates that non-users of PBMAs often exhibit strong negative emotional associations [44], our study demonstrates that even among consumers who do not reject PBMAs, emotional barriers, such as *disappointed*, *unfulfilled*, or *uninspired*, still persist for certain alternatives.

### 3.3. Sensory Drivers of Liking, Purchase Intent, and Emotions

**Figure 3:**
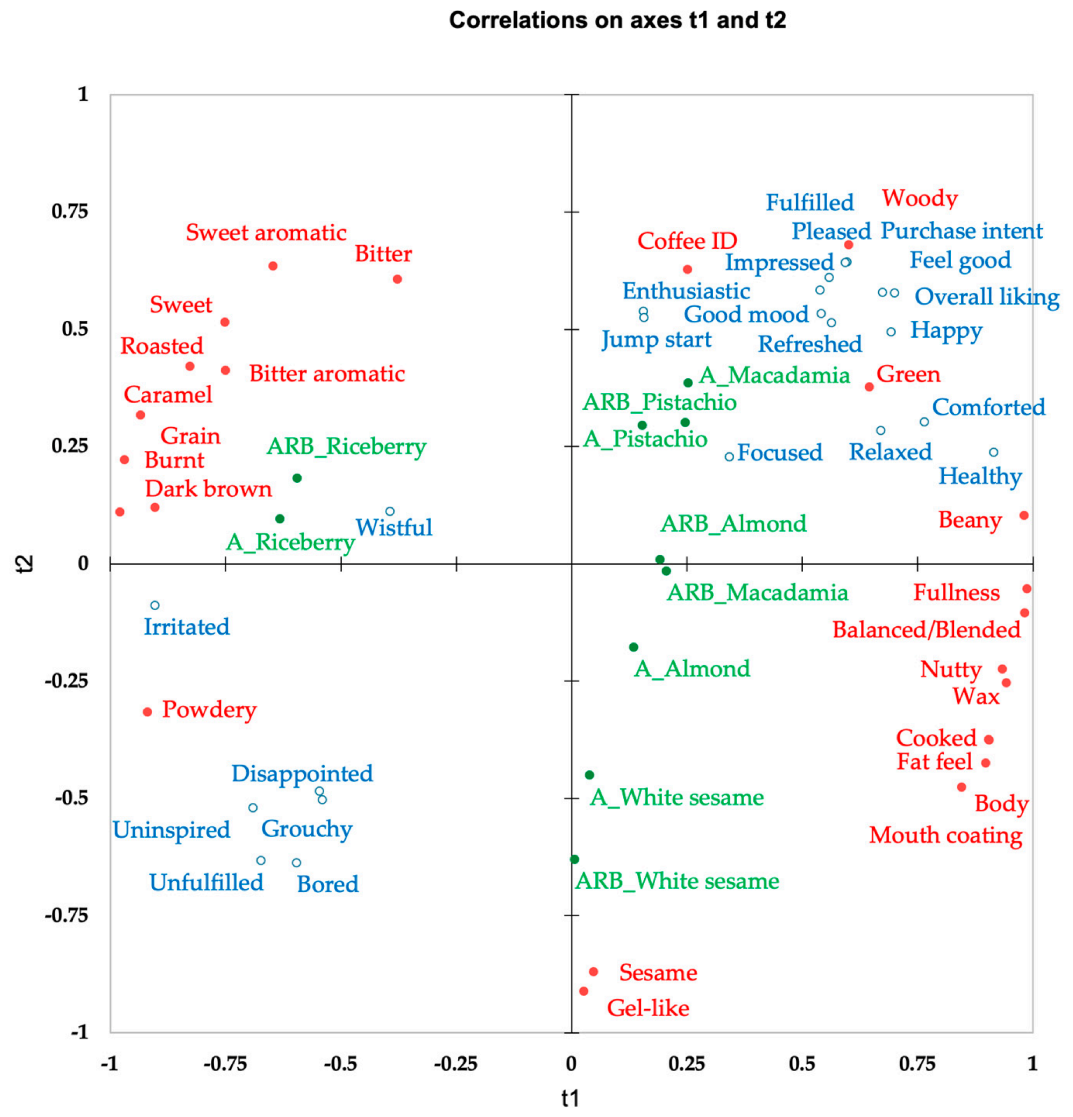
Partial least squares regression (PLSR) biplot illustrating the relationships among chilled sweetened coffee–PBMA samples (●), sensory attributes (●), and consumer responses (●), including overall liking, purchase intent, and emotions. A = Arabica coffee; ARB = Arabica–Robusta blend coffee; PBMA = plant-based milk alternative.

Results from the PLSR analysis identified the sensory drivers of overall liking and purchase intent, and the sensory attributes associated with consumer emotions toward coffee–PBMA samples. The PLSR model with two components explained 85.5% of the variance in the sensory attributes (X, explanatory variables) and 62.1% of the variance in overall liking, top-two-box purchase intent (%), and emotions (proportion of frequency counts) (Y, dependent variables). To evaluate model validation and potential overfitting, full leave-one-out cross-validation (LOO-CV) was applied, yielding a cumulative predictive variance (*Q*^2^) of 38.6% at the second component. The relationships among these variables are visualized in the PLSR biplot (Figure 3). Following the approach described by Lykomitros et al. [45], sensory attributes with standardized beta coefficients greater than 0.05 (in absolute value) and 95% Jackknife confidence intervals excluding zero (Table A3, Appendix A) were considered drivers of liking, purchase intent, and their corresponding emotions.

The results indicated that *nutty*, *beany*, *balanced/blended*, and *fullness* were key sensory drivers of liking and were strongly associated with several positive emotions, including *comforted*, *feel good*, *happy*, and *healthy*. Among these attributes, *beany*, *balanced/blended*, and *fullness* were also positively associated with purchase intent, with *beany* being further linked to additional positive emotions, namely *fulfilled*, *good mood*, *impressed*, *pleased*, and *relaxed*. Furthermore, certain attributes were significantly associated with emotional responses, even though they did not exhibit a strong relationship with overall liking. Specifically, the attribute *woody* was associated with the positive emotions *comforted*, *happy*, *impressed*, and *relaxed*; *bitter taste* with the positive emotion *jump start*; and *coffee ID* with a reduced likelihood of consumers feeling *bored*.

In contrast, the attributes *dark brown* and *powdery* were identified as negative drivers of liking and were associated with negative emotions, including *bored*, *unfulfilled*, and *uninspired*. *Powdery* was also negatively associated with purchase intent and linked to a feeling of *disappointment*, while *dark brown* was further linked to the negative emotion *irritated*. Certain attributes did not show a negative association with liking or purchase intent but were linked to negative emotional responses. Specifically, *burnt* was associated with *irritated*, *sesame* with *disappointed*, and *gel-like* with *bored* and *uninspired*. Interestingly, the attributes *dark brown*, *powdery*, *burnt*, *grain*, *sesame*, and *gel-like* were associated with the emotion term *healthy*.

The positive impact of the *nutty* attribute in this study aligns with the findings of Gorman et al. [7] and Pramudya et al. [9], suggesting that nut-like notes are widely appreciated in both plant-based milk and coffee-milk matrices. Furthermore, the significance of *balanced/blended* (flavor harmony) and *fullness* (robust flavor foundation) as key drivers of liking and positive emotions aligns with the observations of Pinsuwan et al. [23] in brewed black coffee. This consistency, underpinned by the use of identical descriptors and definitions, indicates that a unified sensory experience and a well-rounded flavor foundation represent fundamental factors for consumer satisfaction across different coffee matrices.

A notable point of divergence specifically observed among Thai consumers in this study was the positive role of the *beany* attribute. While the present findings identified *beany* as a driver of liking, purchase intent, and multiple positive emotions, it is frequently cited as a negative attribute in the existing literature. For instance, Gorman et al. [7] and Patabandige et al. [15] both reported that beany or bean-like flavors acted as detractors from liking in Western and Westernized contexts, where these notes are often perceived as off-flavors that must be masked to mimic cow milk [3]. This divergence highlights the critical influence of cultural differences and product familiarity [15]. Many Asian cultures have a long-standing tradition of consuming legume-based products, such as soy milk [46], leading to a higher cultural familiarity with these notes [47]. This is well reflected in our participant profile, where 91% of the consumers reported prior experience with PBMAs, particularly soy milk. Such a high level of pre-existing familiarity suggests that for this specific group of Thai consumers, a beany note does not represent a sensory defect but rather a familiar and authentic characteristic of plant-based beverages that may enhance emotional and hedonic evaluations. Additionally, this positive acceptance might be linked to the specific sensory matrix and flavor intensity of the samples used in this study, where the beany note appeared to be well-balanced rather than overpowering. Consequently, while these findings provide valuable insights into consumers with established experience in PBMAs and specific beverage formulations, they should not be overgeneralized to populations with lower familiarity, different cultural backgrounds, or product categories with different flavor profiles.

Furthermore, the potential impact of sweetness across the formulations warrants a critical discussion. In previous coffee–PBMA studies, sweetness has consistently been identified as a positive driver of liking among both Western [7] and Asian consumers [8]. Given that sugar was added to all formulations in this study to meet consumer preferences, sweetness might be expected to play a similar confounding role. However, our PLSR analysis presents a meaningful divergence; the standardized beta coefficients and confidence intervals (detailed in Table A3, Appendix A) clearly demonstrate that sweetness had no significant effect on overall liking, purchase intent, or emotional responses. This statistical outcome is empirically illustrated by the coffee–riceberry milk formulations; despite possessing higher sweetness intensity (Table 3) due to their commercial sugar content, they actually received lower liking scores than did the other samples (Table 4). Consequently, these findings indicate that while a baseline sweetness is essential for palatability, it did not act as a confounding factor that masked consumer judgment. Instead, consumer preferences and emotional profiling in this specific matrix were genuinely driven by the unique flavor and textural characteristics of the respective PBMAs rather than sweetness alone.

The negative influence of *dark brown* and *powdery* attributes highlights key sensory barriers in coffee–PBMA matrices. The *dark brown* note potentially serves as a sensory cue for overheating and overprocessing, which may detract from the natural quality expected by consumers. Furthermore, the aversion to a *powdery* texture aligns with the findings of Chung et al. [8], who identified creamy and smooth textures as essential drivers of preference for coffee–PBMA beverages. Crucially, these “processed” cues—along with the *burnt* and *gel-like* attributes—further diminished consumers’ association with the emotion term *healthy*, suggesting that these sensory defects might be indirectly linked to a perceived reduction in nutritional and natural integrity.

From a practical standpoint, the findings of this study provide a strategic framework for the plant-based coffee beverage sector. Product developers can utilize these sensory and emotional profiles to optimize formulations and processing methods that better align with consumer expectations. Simultaneously, marketers can leverage these insights to develop branding and communication strategies that can better resonate with the cultural and emotional drivers of their target market.

## 4. Conclusions

This study demonstrates that the sensory profiles and Thai consumer acceptance of chilled sweetened coffee–PBMA beverages are primarily determined by the choice of PBMA, whereas the two evaluated coffee types (100% Arabica and the 70:30 w/w Arabica–Robusta blend) exert only a marginal influence. Macadamia and pistachio milk emerged as promising alternatives, exhibiting significantly (*p* ≤ 0.05) higher overall acceptance and purchase intent than the remaining alternatives. Their association with distinct positive emotional profiles further highlights the potential viability of these alternatives in the tested plant-based coffee market. Sensory attributes, including *nutty*, *beany*, *balanced/blended*, and *fullness*, were identified as the primary factors that foster consumer liking and positive emotional engagement. In contrast, the *dark brown* flavor and *powdery* texture serve as major sensory barriers, linked to a decline in consumer acceptance and the elicitation of negative emotions. Furthermore, this study provides a foundational sensory baseline for riceberry and white sesame milk in coffee applications. Despite their lower acceptance scores, documenting these novel bases is critical for the development of regionally diverse PBMAs. In summary, the insights gained from this research may assist product developers in optimizing coffee–PBMA formulations by focusing on key exploratory sensory drivers and avoiding undesirable attributes, while allowing marketers to leverage cultural and emotional cues to better resonate with the target market.

It should be noted that the findings of this study are most applicable to chilled sweetened coffee beverages prepared with the specific commercial PBMAs tested and evaluated by the surveyed cohort of Thai milk coffee consumers. Furthermore, the consumer panel exhibited a gender imbalance (72% female, 28% male), which may limit the generalizability of the findings across all genders; therefore, future studies should use larger, more demographically balanced consumer cohorts. Additionally, while this study established a comprehensive sensory-consumer framework, the underlying physico-chemical properties of the coffee–PBMA systems were not evaluated. Future research incorporating chemical parameters (such as pH and buffering capacity) and physical parameters (such as total solids, total soluble solids, emulsion stability, colloidal stability, foaming properties, viscosity, rheological behavior, and particle size), alongside both visual and objective color assessments (e.g., CIE L*a*b*), is recommended to fully elucidate the structural mechanisms that drive flavor and mouthfeel perceptions. Finally, future investigations into direct comparisons with traditional oat, soy, and dairy milk could strengthen the practical relevance of these findings.

## Figures and Tables

**Table 1:** The 25 emotion terms and corresponding Thai translations used in the study.

Emotion Terms (Thai Translation)			
Active (กระฉับกระเฉง) ^a,b,c,d^	Enthusiastic (กระตือรือร้น) ^a,b^	Happy (มีความสุข) ^a,b,c^	Refreshed (สดชื่น) ^a,c^
Alert (ตื่นตัว) ^a,c^	Feel good (รู้สึกดี) ^a^	Healthy (สุขภาพดี) ^c^	Relaxed (ผ่อนคลาย) ^a,b,d^
Awake (ตื่น) ^a,d^	Focused (มีสมาธิ) ^c^	Impressed (ประทับใจ) ^a^	Unfulfilled (ไม่เติมเต็ม) ^a,b,c^
Bored (เบื่อ/เซ็ง) ^a,b,d^	Fulfilled (เติมเต็ม) ^c,d^	Irritated (รำคาญ/โกรธ/โมโห) ^b^	Uninspired (ไม่สร้างแรงบันดาลใจ) ^c^
Comforted (สบาย) ^b,c,d^	Good mood (อารมณ์ดี) ^a^	Jump start (พร้อมทำงาน) ^a,d^	Vigorous (กระปรี้กระเปร่า) ^a^
Disappointed (ผิดหวัง) ^a,d^	Grouchy (หงุดหงิด) ^a,d^	Pleased (พอใจ) ^a,b,d^	Wistful (โหยหา) ^a^
Energetic (มีแรง/มีพลัง) ^a,b,c,d^			

^a^ Terms from the study by Pinsuwan et al. [23]. ^b^ Terms from the EsSense Profile^TM^ (ESP) [26]. ^c^ Terms from the WellSense Profile^TM^ [27]. ^d^ Terms from the Coffee-Drinking Experience (COE) Profile [25].

**Table 2:** Attributes, definitions, references, and intensities for evaluating sensory characteristics of chilled sweetened coffee–PBMA samples.

Attribute	Definition		References and Their Intensities
***Flavor***			
Coffee identity (ID) ^b^	A distinctly roasted brown, slightly bitter aromatic characteristic of brewed coffee. Additional descriptors may/may not include woody, oily, acidic, and full-bodied, and these notes may occur at varying intensities.		Nescafé Latte coffee = 4.0 Arabus Latte coffee = 8.0
Roasted ^b,e,f^	The dark brown impression characteristic of foods cooked to a high temperature by dry heat. It does not include bitter or burnt notes.		Tong Garden salted peanuts = 7.0
Brown ^g^	A rich, full brown aromatic of foods heated to become darker in color. It may be related to other aromatics such as roasted and baked.		SW pinto beans = 5.0
Sweet aromatic ^c^	An aromatic associated with the impression of a sweet substance.		Mitr Phol brown sugar 100 g/L brown sugar solution = 3.5 Brown sugar (pure) = 5.5 (only for smelling)
Nutty ^a,c,d,e^	A combination of slightly sweet, brown, woody, oily, and musty aromatics commonly associated with nuts and grains.		Dr. Green wheat germ = 7.5
Burnt ^b,e^	The dark brown carbon impression of an over-cooked or over-roasted product that can be sharp, bitter and sour.		Nescafé Red Cup Expresso Roast instant coffee 1 g coffee in 200 mL water = 8.0 10 g coffee in 200 mL water = 11.0
Woody ^a,c^	The sweet, brown, musty, dark aromatics associated with a bark of a tree.		Heritage premium shelled walnuts = 4.0
Bitter aromatic ^b^	The perception of bitter aromatic of coffee.		Nescafé Red Cup Expresso Roast instant coffee 0.5 g coffee in 200 mL water = 8.0 1 g coffee in 200 mL water = 11.0
Cooked ^g^	An aromatic impression associated with grains, nuts, beans or legumes that have been gently heated or boiled.		Dice brand instant rice 5 g rice in 350 mL hot water Steep for 7 min and strain to obtain only the liquid = 4.0
Beany ^c,d,e,f^	A combination of green, musty/earthy, and musty/dusty aromatics associated with beans and bean products.		Doi Kham soybean powder = 8.0
Dark brown ^g^	An aromatic impression associated with foods that have been heated to the point of being almost burnt.		Hershey’s chocolate syrup 5 g syrup in 200 mL water = 5.5
Wax ^g^	A combination of sweet and petroleum jelly aromatics associated with beeswax.		Peking milk flavored coated wafer filled with cream (0.5 in^3^) = 3.0
Caramel ^e,f^	A sweet aromatic of sugar that has been heated until its color turns into golden brown.		Werther’s original chewy toffee (½ piece) = 8.0
Green ^c^	A sharp and slightly pungent aromatic associated with green plants/vegetables such as parsley, spinach, pea pod, etc.		Ezygo edamame = 3.5
Grain ^d^	Aromatics associated with the overall impression of grains such as corn, wheat, oat, etc.		4Care cereal drink (oat, jasmine brown rice germ and wheat germ) = 8.0
Sesame ^g^	A nutty, roasted, dry, woody, musty and sweet aromatic characteristic of heat-treated sesame seeds.		Sesabac roasted and ground white sesame 3.5 g sesame in 200 mL water = 4.0
Sweet taste ^c,d,e,f^	The fundamental taste factor associated with a sugar solution.		20 g/L sucrose solution = 2.0 50 g/L sucrose solution = 5.0 100 g/L sucrose solution = 10.0
Bitter taste ^b,c,d,e,f^	The fundamental taste factor associated with a caffeine solution.		0.5 g/L caffeine solution = 2.0
Astringent ^b^	A drying, puckering, or tingling sensation on the surface and/or edge of the tongue and mouth.		0.3 g/L alum solution = 1.5
Balanced/Blended ^b,c^	The melding of individual sensory notes such that the product presents a unified overall sensory experience as opposed to spikes or individual notes.		UCC Tokyo Cuppa Latte coffee = 4.0 Arabus Latte coffee = 9.5
Fullness ^b,c^	The foundation of flavor notes that give substance to the product. The perception of robust flavor that is rounded with body		UCC Tokyo Cuppa Latte coffee = 3.0 Arabus Latte coffee = 9.0
***Texture***			
Gel-like ^g^	A soft, smooth and elastic texture of the sample, closely resembling the texture of a gel.		4Care cereal drink (oat, jasmine brown rice germ and wheat germ) = 3.5
Body ^g^	The perception of sample’s density when it is pressed between tongue and palate.		Meiji Gold Maxx pasteurized milk = 7.0 F&N Magnolia whipping cream = 9.0 Anchor whipping cream = 14.0
Powdery ^g^	The perception of small particles distributed within the sample.		Doi Kham soybean powder 2 g in 100 mL water = 4.0
Fat feel ^g^	The perception of fats in the sample.		Meiji pasteurized milk = 6.0
Mouth coating ^g^	The perception of having a starch or fat coating in the oral cavity after sample swallowing.		Meiji pasteurized milk = 6.0

PBMA = plant-based milk alternative. ^a^ Attribute from the study by Pinsuwan et al. [23]. ^b^ Attribute from the study by Pinsuwan et al. [23] with modification of reference samples. ^c^ Attribute from the study by Chambers et al. [24] with modification of reference samples. ^d^ Attribute from the study by Pramudya et al. [9]. ^e^ Attribute from the study by Gorman et al. [7]. ^f^ Attribute from the study by Chung et al. [8]. ^g^ Newly added attribute in the current study.

**Table 3:** Mean intensity scores for sensory attributes of chilled sweetened coffee–PBMA samples prepared from 100% ground Arabica (A) or the 70:30 (w/w) Arabica–Robusta blend (ARB), each combined with almond, pistachio, macadamia, white sesame, or riceberry milk ^#^.

Attributes	Cluster 1			Cluster 2							Cluster 3	
	A– Riceberry	ARB– Riceberry		A– Almond	A– Pistachio	A– Macadamia	ARB– Almond	ARB– Pistachio	ARB– Macadamia		A– White Sesame	ARB– White Sesame
**Flavor**												
Coffee ID	6.18 ± 0.60 ^b,c^	6.36 ± 0.59 ^a,b,c^		6.11 ± 0.11 ^c^	6.40 ± 0.03 ^a,b,c^	6.57 ± 0.30 ^a^	6.25 ± 0.12 ^b,c^	6.44 ± 0.16 ^a,b^	6.24 ± 0.18 ^b,c^		6.33 ± 0.31 ^a,b,c^	6.19 ± 0.20 ^b,c^
Roasted	4.44 ± 0.55 ^a^	4.50 ± 0.39 ^a^		3.89 ± 0.31 ^b,c^	4.03 ± 0.12 ^b,c^	4.03 ± 0.35 ^b,c^	3.80 ± 0.20 ^c^	4.21 ± 0.49 ^a,b^	3.75 ± 0.35 ^c^		3.96 ± 0.57 ^b,c^	3.89 ± 0.00 ^b,c^
Brown ^ns^	6.36 ± 0.27	6.14 ± 0.12		6.25 ± 0.12	6.39 ± 0.08	6.33 ± 0.00	6.17 ± 0.16	6.22 ± 0.00	6.11 ± 0.00		6.14 ± 0.43	6.03 ± 0.04
Sweet aromatic	4.29 ± 0.05 ^a^	4.12 ± 0.06 ^a^		3.69 ± 0.12 ^d,e,f^	3.86 ± 0.12 ^c,d^	3.97 ± 0.12 ^b,c^	3.79 ± 0.14 ^c,d,e^	3.81 ± 0.20 ^c,d,e^	3.96 ± 0.02 ^b,c^		3.57 ± 0.13 ^f^	3.61 ± 0.08 ^e,f^
Nutty	2.22 ± 0.08 ^d^	2.14 ± 0.20 ^d^		4.28 ± 0.39 ^b,c^	4.22 ± 0.00 ^b,c^	4.47 ± 0.12 ^a,b^	4.03 ± 0.12 ^c^	4.02 ± 0.34 ^c^	4.79 ± 0.25 ^a^		4.52 ± 0.02 ^a,b^	4.25 ± 0.27 ^b,c^
Burnt	3.58 ± 0.12 ^a^	3.39 ± 0.79 ^a^		2.50 ± 0.16 ^c,d,e^	2.72 ± 0.31 ^b,c^	2.67 ± 0.63 ^b,c,d^	2.36 ± 0.27 ^d,e^	2.75 ± 0.67 ^b,c^	2.28 ± 0.24 ^e^		2.94 ± 0.86 ^b^	2.69 ± 0.04 ^b,c,d^
Woody	1.78 ± 0.08 ^c,d^	1.93 ± 0.09 ^b,c,d^		2.10 ± 0.09 ^a,b,c^	2.39 ± 0.16 ^a^	2.21 ± 0.13 ^a,b^	2.11 ± 0.31 ^a,b,c^	2.09 ± 0.52 ^a,b,c,d^	2.00 ± 0.16 ^b,c,d^		1.84 ± 0.14 ^b,c,d^	1.72 ± 0.24 ^d^
Bitter aromatic	3.25 ± 0.27 ^a,b^	3.50 ± 0.24 ^a^		2.90 ± 0.06 ^b,c,d^	2.89 ± 0.00 ^b,c,d^	2.94 ± 0.39 ^b,c,d^	2.76 ± 0.05 ^c,d^	3.09 ± 0.34 ^b,c^	2.62 ± 0.12 ^d^		3.03 ± 0.67 ^b,c^	2.61 ± 0.16 ^d^
Cooked	0.22 ± 0.31 ^e^	0.56 ± 0.16 ^d^		1.52 ± 0.13 ^a,b,c^	1.50 ± 0.00 ^a,b,c^	1.44 ± 0.08 ^a,b,c^	1.36 ± 0.04 ^b,c^	1.31 ± 0.20 ^c^	1.54 ± 0.06 ^a,b,c^		1.64 ± 0.12 ^a,b^	1.67 ± 0.16 ^a^
Beany	0.50 ± 0.24 ^f^	0.36 ± 0.04 ^f^		3.58 ± 0.27 ^c^	4.47 ± 0.04 ^a^	3.81 ± 0.04 ^b,c^	3.75 ± 0.12 ^b,c^	3.56 ± 0.00 ^c^	4.06 ± 0.24 ^b^		3.00 ± 0.55 ^d^	2.42 ± 0.27 ^e^
Dark brown	2.58 ± 0.12 ^a^	2.53 ± 0.12 ^a^		0.54 ± 0.09 ^c^	0.88 ± 0.30 ^b,c^	0.47 ± 0.67 ^c^	0.75 ± 0.20 ^b,c^	0.89 ± 0.31 ^b,c^	0.67 ± 0.63 ^b,c^		0.89 ± 0.94 ^b,c^	1.06 ± 0.31 ^b^
Wax	0.00 ± 0.00 ^d^	0.00 ± 0.00 ^d^		1.19 ± 0.35 ^a,b^	1.34 ± 0.06 ^a^	0.92 ± 0.24 ^c^	0.92 ± 0.04 ^c^	1.14 ± 0.40 ^a,b,c^	1.25 ± 0.04 ^a,b^		1.18 ± 0.02 ^a,b^	1.07 ± 0.05 ^b,c^
Caramel	2.61 ± 0.24 ^b^	2.84 ± 0.22 ^a^		0.50 ± 0.00 ^d,e^	0.69 ± 0.04 ^c^	0.58 ± 0.04 ^c,d^	0.47 ± 0.12 ^d,e^	0.47 ± 0.20 ^d,e^	0.58 ± 0.12 ^c,d^		0.39 ± 0.08 ^e,f^	0.28 ± 0.08 ^f^
Green	0.00 ± 0.00 ^b^	0.00 ± 0.00 ^b^		0.28 ± 0.39 ^b^	0.83 ± 0.86 ^a^	0.61 ± 0.36 ^a^	0.90 ± 0.35 ^a^	0.72 ± 0.24 ^a^	0.00 ± 0.00 ^b^		0.18 ± 0.25 ^b^	0.28 ± 0.16 ^b^
Grain	2.17 ± 0.08 ^a^	2.25 ± 0.04 ^a^		0.00 ± 0.00 ^b^	0.00 ± 0.00 ^b^	0.00 ± 0.00 ^b^	0.00 ± 0.00 ^b^	0.00 ± 0.00 ^b^	0.00 ± 0.00 ^b^		0.00 ± 0.00 ^b^	0.00 ± 0.00 ^b^
Sesame	0.00 ± 0.00 ^c^	0.00 ± 0.00 ^c^		0.00 ± 0.00 ^c^	0.00 ± 0.00 ^c^	0.00 ± 0.00 ^c^	0.00 ± 0.00 ^c^	0.00 ± 0.00 ^c^	0.00 ± 0.00 ^c^		1.47 ± 0.27 ^b^	2.11 ± 0.08 ^a^
Sweet taste	7.83 ± 0.00 ^a^	7.62 ± 0.04 ^b^		6.35 ± 0.10 ^g^	6.97 ± 0.20 ^c,d^	7.00 ± 0.08 ^c^	6.78 ± 0.08 ^d,e^	6.72 ± 0.00 ^e,f^	7.06 ± 0.08 ^c^		6.44 ± 0.00 ^g^	6.56 ± 0.24 ^f,g^
Bitter taste	1.40 ± 0.05 ^a,b,c^	1.53 ± 0.04 ^a^		1.43 ± 0.34 ^a,b^	1.33 ± 0.08 ^b,c^	1.40 ± 0.22 ^a,b,c^	1.23 ± 0.02 ^b,c^	1.54 ± 0.31 ^a^	1.27 ± 0.11 ^b,c^		1.29 ± 0.14 ^b,c^	1.21 ± 0.09 ^c^
Astringent ^ns^	0.66 ± 0.07	0.69 ± 0.01		0.62 ± 0.06	0.68 ± 0.05	0.56 ± 0.16	0.61 ± 0.05	0.69 ± 0.03	0.63 ± 0.05		0.73 ± 0.14	0.67 ± 0.10
Balanced/Blended	6.25 ± 0.04 ^c^	6.22 ± 0.31 ^c^		7.97 ± 0.59 ^a,b^	7.83 ± 0.08 ^a,b^	8.14 ± 0.12 ^a^	8.22 ± 0.16 ^a^	7.83 ± 0.39 ^a,b^	8.01 ± 0.02 ^a,b^		7.83 ± 0.24 ^a,b^	7.64 ± 0.20 ^b^
Fullness	5.53 ± 0.20 ^c^	5.54 ± 0.10 ^c^		6.94 ± 0.16 ^b^	7.19 ± 0.04 ^a,b^	7.36 ± 0.20 ^a^	7.06 ± 0.08 ^a,b^	7.19 ± 0.27 ^a,b^	7.19 ± 0.04 ^a,b^		7.11 ± 0.00 ^a,b^	6.86 ± 0.12 ^b^
**Texture**												
Gel-like	0.17 ± 0.24 ^e^	0.53 ± 0.35 ^d^		1.03 ± 1.06 ^c^	0.11 ± 0.16 ^e^	0.15 ± 0.21 ^e^	0.42 ± 0.51 ^d,e^	0.23 ± 0.06 ^d,e^	0.31 ± 0.43 ^d,e^		2.07 ± 0.03 ^a^	1.69 ± 0.12 ^b^
Body	4.97 ± 0.20 ^d^	5.08 ± 0.20 ^d^		7.82 ± 0.46 ^b^	7.63 ± 0.13 ^b,c^	7.61 ± 0.11 ^b,c^	7.53 ± 0.47 ^b,c^	7.43 ± 0.38 ^c^	7.86 ± 0.12 ^b^		8.19 ± 0.12 ^a^	8.36 ± 0.16 ^a^
Powdery	2.10 ± 0.14 ^a^	2.27 ± 0.02 ^a^		0.86 ± 0.27 ^c^	0.57 ± 0.02 ^c^	0.58 ± 0.04 ^c^	0.54 ± 0.22 ^c^	0.66 ± 0.25 ^c^	0.57 ± 0.10 ^c^		1.56 ± 0.39 ^b^	1.58 ± 0.20 ^b^
Fat feel	1.75 ± 0.12 ^f^	1.96 ± 0.02 ^f^		4.08 ± 0.67 ^b,c,d^	3.99 ± 0.14 ^c,d,e^	4.15 ± 0.02 ^b,c^	3.69 ± 0.04 ^e^	3.80 ± 0.16 ^d,e^	4.22 ± 0.16 ^b,c^		4.31 ± 0.04 ^a,b^	4.56 ± 0.08 ^a^
Mouth coating	3.19 ± 0.12 ^c^	3.14 ± 0.04 ^c^		4.17 ± 0.55 ^a,b^	3.99 ± 0.14 ^b^	4.17 ± 0.27 ^a,b^	3.94 ± 0.24 ^b^	3.85 ± 0.18 ^b^	4.31 ± 0.12 ^a^		4.34 ± 0.06 ^a^	4.36 ± 0.12 ^a^

PBMA = plant-based milk alternative. ^#^ Samples are arranged into columns according to their respective cluster designations based on Hierarchical Cluster Analysis (HCA) results. Scores represent the mean of two replications evaluated by nine panelists. ^a–g^ Scores in the same row with different superscripts are significantly different (*p* ≤ 0.05), based on Duncan’s multiple range test. ^ns^ Scores in the same row are not significantly different (*p* > 0.05).

**Table 4:** Overall liking scores and purchase intent of chilled sweetened coffee–PBMA samples prepared from 100% ground Arabica (A) or the 70:30 (w/w) Arabica–Robusta blend (ARB), each combined with almond, pistachio, macadamia, white sesame, or riceberry milk.

Coffee–PBMA Samples	Overall Liking Scores	Top-Two-Box Purchase Intent (%)
A–Macadamia	7.1 ± 1.2 ^a^	84 ^a^
ARB–Pistachio	6.7 ± 1.4 ^b^	69 ^a,b^
A–Pistachio	6.5 ± 1.5 ^b^	62 ^b,c^
ARB–Macadamia	6.4 ± 1.7 ^b^	62 ^b,c^
ARB–Almond	5.6 ± 1.7 ^c^	36 ^d^
A–Almond	5.5 ± 1.8 ^c,d^	42 ^c,d^
A–White sesame	5.5 ± 1.7 ^c,d^	28 ^d^
ARB–White sesame	5.4 ± 1.9 ^c,d^	35 ^d^
A–Riceberry	5.2 ± 1.9 ^d^	31 ^d^
ARB–Riceberry	5.0 ± 2.0 ^d^	32 ^d^

PBMA = plant-based milk alternative. Liking scores were the average values of 100 consumers. Top-Two-box purchase intent represents the percentage of consumers selecting “definitely would purchase” and “probably would purchase”. ^a–d^ Liking scores and Top-Two-Box purchase intent with different superscripts are significantly different (*p* ≤ 0.05).

## Data Availability

The original contributions presented in this study are included in the article. Further inquiries can be directed to the corresponding author.

## Appendix A

**Table A1:** Ingredients and nutrition information as declared on the packaging of the plant-based milk alternatives (PBMAs) used in the study.

Parameter	Almond Milk	Macadamia Milk	Pistachio Milk	White Sesame Milk	Riceberry Milk
Ingredients	Almond milk (95%) Sunflower seeds (5%)	Macadamia milk (95%) Sunflower seeds (5%)	Pistachio milk (95%) Sunflower seeds (5%)	White sesame milk (100%)	Riceberry milk (94%) Sugar (4%) Sunflower seed oil (2%)
Serving size (mL)	180	180	180	200	180
Nutritional values (per serving size)					
Total energy (Kcal)	60	60	60	110	80
Energy from fat (Kcal)	45	45	45	60	25
Total fat (g)	5	5	5	7	3
Saturated fat (g)	0.5	0.5	1	1	0
Trans fat (g)	0	0	0	-	-
Monounsaturated fat (g)	-	-	1.5	3	-
Polyunsaturated fat (g)	-	-	2.5	2.5	-
Cholesterol (mg)	0	0	0	0	0
Protein (g)	2	1	1	3	<1
Total carbohydrate (g)	1	3	3	8	12
Dietary fiber (g)	0	1	1	2	<1
Sugars (g)	0	1	1	0	7
Sodium (mg)	10	50	50	35	40

“-”: Data not declared on the product packaging.

**Table A2:** Proportion of consumers selecting each emotion term for chilled sweetened coffee–PBMA samples prepared from 100% ground Arabica (A) or the 70:30 (w/w) Arabica–Robusta blend (ARB), each combined with almond, pistachio, macadamia, white sesame, or riceberry milk.

Emotions	A– Macadamia	ARB– Pistachio	A– Pistachio	ARB– Macadamia	ARB– Almond	A– Almond	A– White Sesame	ARB– White Sesame	A– Riceberry	ARB– Riceberry
Comforted	0.260 ^abc^	0.330 ^a^	0.200 ^abcd^	0.290 ^ab^	0.180 ^abcd^	0.200 ^abcd^	0.120 ^bcd^	0.180 ^abcd^	0.060 ^d^	0.100 ^cd^
Enthusiastic	0.200 ^a^	0.080 ^a^	0.190 ^a^	0.170 ^a^	0.070 ^a^	0.100 ^a^	0.090 ^a^	0.090 ^a^	0.100 ^a^	0.150 ^a^
Feel good	0.480 ^a^	0.340 ^abc^	0.340 ^abc^	0.360 ^ab^	0.240 ^bcd^	0.160 ^cd^	0.220 ^bcd^	0.140 ^d^	0.130 ^d^	0.140 ^d^
Focused	0.090 ^ab^	0.180 ^a^	0.090 ^ab^	0.100 ^ab^	0.070 ^ab^	0.100 ^ab^	0.050 ^b^	0.110 ^ab^	0.070 ^ab^	0.070 ^ab^
Fulfilled	0.260 ^a^	0.180 ^ab^	0.170 ^ab^	0.130 ^ab^	0.120 ^ab^	0.100 ^b^	0.070 ^b^	0.090 ^b^	0.090 ^b^	0.060 ^b^
Good mood	0.400 ^a^	0.200 ^bc^	0.240 ^abc^	0.310 ^ab^	0.160 ^bc^	0.130 ^c^	0.120 ^c^	0.140 ^bc^	0.130 ^c^	0.120 ^c^
Happy	0.270 ^a^	0.250 ^ab^	0.190 ^abc^	0.180 ^abc^	0.130 ^abcd^	0.060 ^cd^	0.120 ^bcd^	0.090 ^cd^	0.070 ^cd^	0.020 ^d^
Healthy	0.260 ^a^	0.290 ^a^	0.260 ^a^	0.230 ^ab^	0.260 ^a^	0.250 ^a^	0.140 ^abc^	0.190 ^abc^	0.080 ^bc^	0.070 ^c^
Impressed	0.320 ^a^	0.280 ^a^	0.190 ^abc^	0.200 ^ab^	0.070 ^bc^	0.090 ^bc^	0.100 ^bc^	0.080 ^bc^	0.100 ^bc^	0.040 ^c^
Jump start	0.330 ^a^	0.180 ^abc^	0.290 ^ab^	0.170 ^bc^	0.150 ^bc^	0.120 ^c^	0.210 ^abc^	0.160 ^bc^	0.220 ^abc^	0.170 ^bc^
Pleased	0.390 ^a^	0.330 ^ab^	0.220 ^abc^	0.260 ^abc^	0.190 ^bc^	0.180 ^bc^	0.130 ^c^	0.150 ^c^	0.170 ^bc^	0.110 ^c^
Refreshed	0.510 ^a^	0.330 ^abc^	0.260 ^bcd^	0.370 ^ab^	0.210 ^bcd^	0.210 ^bcd^	0.140 ^d^	0.210 ^bcd^	0.180 ^cd^	0.140 ^d^
Relaxed	0.200 ^abc^	0.280 ^a^	0.160 ^abc^	0.240 ^ab^	0.190 ^abc^	0.080 ^c^	0.140 ^abc^	0.140 ^abc^	0.090 ^bc^	0.060 ^c^
Wistful	0 ^a^	0 ^a^	0.050 ^a^	0.050 ^a^	0 ^a^	0 ^a^	0.010 ^a^	0.020 ^a^	0.050 ^a^	0.030 ^a^
Bored	0.010 ^c^	0.020 ^c^	0.040 ^bc^	0.030 ^c^	0.070 ^abc^	0.120 ^abc^	0.080 ^abc^	0.180 ^a^	0.110 ^abc^	0.150 ^ab^
Disappointed	0.020 ^e^	0.080 ^cde^	0.070 ^de^	0.110 ^bcde^	0.240 ^ab^	0.270 ^a^	0.170 ^abcde^	0.200 ^abcd^	0.230 ^abc^	0.240 ^ab^
Grouchy	0.020 ^a^	0.020 ^a^	0.030 ^a^	0.060 ^a^	0.080 ^a^	0.050 ^a^	0.050 ^a^	0.110 ^a^	0.060 ^a^	0.120 ^a^
Irritated	0.010 ^b^	0.040 ^ab^	0.040 ^ab^	0.030 ^b^	0.030 ^b^	0.060 ^ab^	0.060 ^ab^	0.050 ^ab^	0.090 ^ab^	0.130 ^a^
Unfulfilled	0.060 ^d^	0.110 ^bcd^	0.120 ^abcd^	0.080 ^cd^	0.200 ^abcd^	0.240 ^abc^	0.260 ^ab^	0.290 ^a^	0.290 ^a^	0.270 ^ab^
Uninspired	0.020 ^d^	0.040 ^bcd^	0.070 ^abcd^	0.090 ^abcd^	0.030 ^cd^	0.180 ^a^	0.120 ^abcd^	0.170 ^ab^	0.200 ^a^	0.160 ^abc^

Cochran’s Q test was used to identify significant differences among samples. ^a–e^ Different superscripts indicate significant differences based on Sheskin post hoc comparisons (*p* ≤ 0.05).

**Table A3:** Standardized beta coefficients (β) and 95% confidence intervals (CIs) for the relationships between sensory attributes and consumer responses (overall liking, purchase intent, and emotions) derived from the PLSR model. Bold values indicate significant associations based on jackknife 95% confidence intervals that exclude zero.

Sensory Attributes	Overall Liking			Purchase Intent			Comforted		
	β	Lower Bound	Upper Bound	β	Lower Bound	Upper Bound	β	Lower Bound	Upper Bound
Coffee ID	0.184	−0.063	0.430	0.193	−0.120	0.505	0.123	−0.117	0.362
Roasted	0.017	−0.047	0.081	0.026	−0.056	0.108	−0.006	−0.075	0.063
Sweet aromatic	0.081	−0.091	0.253	0.092	−0.109	0.293	0.038	−0.109	0.186
Nutty	**0.054**	0.024	0.084	**0.048**	0.013	0.084	**0.054**	0.016	0.091
Burnt	−0.037	−0.093	0.019	−0.031	−0.089	0.027	−0.043	−0.083	−0.003
Woody	0.130	−0.019	0.279	0.133	−0.031	0.296	**0.095**	0.004	0.186
Bitter aromatic	−0.001	−0.069	0.067	0.006	−0.061	0.074	−0.016	−0.063	0.031
Cooked	0.012	−0.016	0.041	0.005	−0.025	0.034	0.026	−0.012	0.063
Beany	**0.093**	0.054	0.132	**0.089**	0.048	0.131	**0.079**	0.056	0.102
Dark brown	**−0.065**	−0.122	−0.007	−0.059	−0.123	0.004	**−0.062**	−0.106	−0.018
Wax	0.038	−0.047	0.122	0.031	−0.046	0.109	0.043	−0.014	0.100
Caramel	−0.030	−0.106	0.046	−0.023	−0.097	0.051	−0.039	−0.095	0.017
Green	0.086	−0.023	0.195	0.085	−0.035	0.205	0.069	−0.011	0.150
Grain	−0.047	−0.099	0.006	−0.040	−0.090	0.010	**−0.050**	−0.093	−0.007
Sesame	−0.121	−0.254	0.013	−0.128	−0.279	0.023	−0.076	−0.172	0.019
Sweet taste	0.050	−0.100	0.199	0.060	−0.109	0.229	0.016	−0.102	0.134
Bitter taste	0.075	−0.019	0.168	0.082	−0.044	0.209	0.041	−0.059	0.140
Balanced/Blended	**0.061**	0.015	0.107	0.092	−0.109	0.293	**0.059**	0.026	0.093
Fullness	**0.088**	0.035	0.141	**0.048**	0.013	0.084	**0.077**	0.013	0.140
Gel-like	−0.160	−0.346	0.025	−0.170	−0.406	0.065	−0.103	−0.284	0.079
Body	0.010	−0.042	0.061	0.002	−0.048	0.051	0.024	−0.017	0.066
Powdery	**−0.125**	−0.205	−0.044	**−0.123**	−0.229	−0.018	**−0.099**	−0.180	−0.018
Fat feel	0.027	−0.024	0.078	0.193	−0.120	0.505	0.036	−0.010	0.081
Mouth coating	0.010	−0.027	0.048	0.026	−0.056	0.108	0.023	−0.010	0.057
**Sensory** **Attributes**	**Enthusiastic**			**Feel Good**			**Focused**		
	**β**	**Lower Bound**	**Upper Bound**	**β**	**Lower Bound**	**Upper Bound**	**β**	**Lower Bound**	**Upper Bound**
Coffee ID	0.138	−0.121	0.397	0.182	−0.059	0.423	0.077	−0.252	0.405
Roasted	0.034	−0.017	0.086	0.018	−0.041	0.078	0.004	−0.084	0.092
Sweet aromatic	0.081	−0.090	0.251	0.082	−0.093	0.256	0.031	−0.148	0.211
Nutty	0.019	−0.038	0.075	**0.052**	0.023	0.081	0.025	−0.026	0.077
Burnt	−0.006	−0.076	0.065	−0.036	−0.096	0.025	−0.018	−0.048	0.011
Woody	0.088	−0.130	0.306	0.129	−0.041	0.298	0.056	−0.054	0.166
Bitter aromatic	0.019	−0.035	0.072	0.000	−0.071	0.072	−0.003	−0.029	0.023
Cooked	−0.013	−0.040	0.014	0.011	−0.019	0.041	0.008	−0.022	0.038
Beany	0.048	−0.053	0.149	**0.091**	0.048	0.134	0.042	−0.033	0.116
Dark brown	−0.026	−0.101	0.049	**−0.063**	−0.121	−0.005	−0.030	−0.089	0.028
Wax	0.006	−0.040	0.052	0.036	−0.043	0.116	0.019	−0.029	0.067
Caramel	0.001	−0.035	0.037	−0.029	−0.102	0.045	−0.016	−0.051	0.019
Green	0.050	−0.043	0.144	0.085	−0.016	0.185	0.038	−0.062	0.138
Grain	−0.012	−0.035	0.012	−0.045	−0.090	0.000	−0.023	−0.067	0.021
Sesame	−0.096	−0.279	0.087	−0.120	−0.268	0.028	−0.050	−0.178	0.078
Sweet taste	0.059	−0.082	0.200	0.050	−0.103	0.204	0.018	−0.118	0.154
Bitter taste	0.067	−0.005	0.140	0.075	−0.005	0.155	0.030	−0.109	0.169
Balanced/Blended	0.023	−0.043	0.088	**0.059**	0.017	0.102	0.029	−0.021	0.079
Fullness	0.044	−0.028	0.116	**0.086**	0.044	0.128	0.040	−0.061	0.140
Gel-like	−0.126	−0.343	0.090	−0.160	−0.344	0.025	−0.066	−0.326	0.193
Body	−0.015	−0.046	0.016	0.008	−0.044	0.060	0.007	−0.021	0.035
Powdery	−0.074	−0.205	0.057	**−0.123**	−0.200	−0.045	−0.055	−0.192	0.083
Fat feel	−0.002	−0.035	0.032	0.026	−0.026	0.077	0.014	−0.021	0.049
Mouth coating	−0.013	−0.047	0.020	0.009	−0.033	0.051	0.007	−0.010	0.024
**Sensory** **Attributes**	**Fulfilled**			**Good Mood**			**Happy**		
	**β**	**Lower Bound**	**Upper Bound**	**β**	**Lower Bound**	**Upper Bound**	**β**	**Lower Bound**	**Upper Bound**
Coffee ID	0.192	−0.153	0.536	0.163	−0.185	0.511	0.164	−0.069	0.396
Roasted	0.026	−0.057	0.109	0.020	−0.059	0.098	0.011	−0.051	0.074
Sweet aromatic	0.092	−0.127	0.311	0.076	−0.146	0.298	0.069	−0.085	0.223
Nutty	**0.048**	0.005	0.091	0.043	−0.002	0.088	**0.052**	0.011	0.093
Burnt	−0.030	−0.083	0.022	−0.028	−0.081	0.025	−0.037	−0.089	0.015
Woody	0.132	−0.044	0.308	0.113	−0.091	0.317	**0.118**	0.006	0.229
Bitter aromatic	0.006	−0.057	0.069	0.004	−0.054	0.061	−0.004	−0.068	0.059
Cooked	0.004	−0.025	0.034	0.006	−0.021	0.033	0.015	−0.014	0.044
Beany	**0.089**	0.031	0.147	**0.077**	0.003	0.152	**0.087**	0.040	0.133
Dark brown	−0.059	−0.131	0.013	−0.052	−0.118	0.014	−0.062	−0.124	0.000
Wax	0.031	−0.045	0.108	0.029	−0.023	0.080	0.038	−0.048	0.123
Caramel	−0.023	−0.104	0.059	−0.022	−0.090	0.047	−0.031	−0.109	0.047
Green	0.085	−0.049	0.218	0.073	−0.040	0.187	0.079	−0.027	0.186
Grain	−0.040	−0.100	0.021	−0.036	−0.081	0.008	−0.046	−0.108	0.016
Sesame	−0.128	−0.295	0.039	−0.108	−0.298	0.082	**−0.106**	−0.208	−0.005
Sweet taste	0.060	−0.124	0.243	0.049	−0.136	0.233	0.040	−0.095	0.175
Bitter taste	0.082	−0.052	0.217	0.069	−0.054	0.191	0.064	−0.021	0.150
Balanced/Blended	0.055	−0.004	0.114	0.049	−0.003	0.101	**0.059**	0.005	0.112
Fullness	0.084	−0.003	0.171	0.073	−0.011	0.158	**0.083**	0.014	0.152
Gel-like	−0.170	−0.435	0.095	−0.143	−0.421	0.135	−0.142	−0.313	0.029
Body	0.002	−0.054	0.057	0.004	−0.047	0.054	0.013	−0.038	0.063
Powdery	−0.123	−0.250	0.004	−0.106	−0.243	0.031	**−0.115**	−0.200	−0.029
Fat feel	0.020	−0.037	0.077	0.019	−0.028	0.067	0.028	−0.026	0.083
Mouth coating	0.003	−0.032	0.037	0.004	−0.029	0.038	0.013	−0.025	0.051
**Sensory** **Attributes**	**Healthy**			**Impressed**			**Jump Start**		
	**β**	**Lower Bound**	**Upper Bound**	**β**	**Lower Bound**	**Upper Bound**	**β**	**Lower Bound**	**Upper Bound**
Coffee ID	0.117	−0.055	0.290	0.174	−0.097	0.446	0.135	−0.051	0.321
Roasted	−0.016	−0.057	0.025	0.023	−0.048	0.095	0.033	−0.015	0.082
Sweet aromatic	0.027	−0.110	0.165	0.084	−0.082	0.250	0.078	−0.062	0.219
Nutty	**0.062**	0.041	0.083	0.044	−0.001	0.089	0.018	−0.007	0.044
Burnt	**−0.052**	−0.074	−0.029	−0.028	−0.086	0.031	−0.006	−0.061	0.050
Woody	0.096	−0.028	0.220	**0.120**	0.004	0.236	0.086	−0.051	0.223
Bitter aromatic	−0.024	−0.069	0.020	0.006	−0.060	0.072	0.018	−0.034	0.070
Cooked	0.036	0.001	0.071	0.004	−0.021	0.029	−0.012	−0.039	0.014
Beany	**0.087**	0.050	0.123	**0.081**	0.031	0.130	0.047	−0.007	0.102
Dark brown	**−0.070**	−0.103	−0.038	−0.054	−0.123	0.016	−0.025	−0.084	0.033
Wax	0.052	−0.008	0.113	0.028	−0.054	0.111	0.006	−0.054	0.067
Caramel	−0.049	−0.112	0.015	−0.021	−0.098	0.056	0.000	−0.059	0.060
Green	0.073	−0.006	0.152	0.077	−0.042	0.196	0.050	−0.041	0.140
Grain	**−0.059**	−0.103	−0.015	−0.036	−0.099	0.027	−0.012	−0.055	0.032
Sesame	**−0.071**	−0.195	0.053	**−0.116**	−0.225	−0.008	−0.094	−0.213	0.025
Sweet taste	0.005	−0.110	0.120	0.054	−0.087	0.196	0.057	−0.071	0.185
Bitter taste	0.034	−0.036	0.103	0.075	−0.035	0.184	**0.066**	0.014	0.118
Balanced/Blended	**0.068**	0.041	0.096	0.050	−0.010	0.111	0.022	−0.030	0.075
Fullness	**0.085**	0.046	0.124	0.076	−0.005	0.157	0.043	0.000	0.087
Gel-like	−0.096	−0.248	0.057	−0.154	−0.352	0.043	−0.123	−0.272	0.025
Body	0.034	−0.015	0.084	0.001	−0.046	0.049	−0.015	−0.058	0.029
Powdery	**−0.105**	−0.172	−0.037	**−0.112**	−0.214	−0.009	−0.073	−0.150	0.005
Fat feel	0.045	0.008	0.082	0.018	−0.039	0.075	−0.001	−0.040	0.038
Mouth coating	0.033	0.001	0.065	0.003	−0.032	0.037	−0.013	−0.043	0.017
**Sensory Attributes**	**Pleased**			**Refreshed**			**Relaxed**		
	**β**	**Lower Bound**	**Upper Bound**	**β**	**Lower Bound**	**Upper Bound**	**β**	**Lower Bound**	**Upper Bound**
Coffee ID	0.182	−0.165	0.529	0.160	−0.238	0.557	0.112	−0.091	0.315
Roasted	0.025	−0.059	0.109	0.017	−0.080	0.115	−0.004	−0.059	0.052
Sweet aromatic	0.087	−0.119	0.294	0.073	−0.163	0.308	0.037	−0.083	0.156
Nutty	0.045	−0.009	0.100	0.044	−0.011	0.099	**0.047**	0.001	0.094
Burnt	−0.029	−0.083	0.026	−0.030	−0.076	0.017	−0.037	−0.075	0.001
Woody	0.125	−0.022	0.272	0.112	−0.074	0.298	**0.086**	0.033	0.139
Bitter aromatic	0.006	−0.058	0.071	0.002	−0.056	0.059	−0.013	−0.061	0.035
Cooked	0.004	−0.025	0.033	0.008	−0.022	0.038	0.022	−0.006	0.050
Beany	**0.084**	0.024	0.144	0.078	−0.001	0.157	**0.071**	0.021	0.121
Dark brown	−0.056	−0.133	0.021	−0.054	−0.126	0.019	**−0.055**	−0.107	−0.003
Wax	0.029	−0.047	0.106	0.030	−0.022	0.082	0.038	−0.035	0.110
Caramel	−0.021	−0.103	0.061	−0.023	−0.096	0.049	−0.034	−0.095	0.028
Green	0.080	−0.053	0.213	0.073	−0.057	0.203	0.062	−0.018	0.142
Grain	−0.038	−0.105	0.030	−0.038	−0.094	0.019	−0.044	−0.099	0.011
Sesame	−0.121	−0.267	0.025	−0.106	−0.293	0.082	**−0.070**	−0.131	−0.010
Sweet taste	0.057	−0.113	0.227	0.046	−0.145	0.237	0.016	−0.083	0.116
Bitter taste	0.078	−0.062	0.219	0.066	−0.091	0.223	0.038	−0.038	0.115
Balanced/Blended	0.052	−0.012	0.116	0.050	−0.009	0.109	**0.053**	0.007	0.098
Fullness	0.079	−0.022	0.180	0.074	−0.035	0.183	0.068	−0.005	0.142
Gel-like	−0.161	−0.423	0.101	−0.140	−0.453	0.172	−0.094	−0.245	0.056
Body	0.001	−0.051	0.054	0.006	−0.045	0.056	0.021	−0.019	0.060
Powdery	−0.116	−0.250	0.018	−0.106	−0.264	0.052	**−0.089**	−0.173	−0.005
Fat feel	0.019	−0.044	0.082	0.021	−0.033	0.075	0.031	−0.016	0.078
Mouth coating	0.002	−0.034	0.039	0.006	−0.027	0.040	0.020	−0.013	0.053
**Sensory** **Attributes**	**Wistful**			**Bored**			**Disappointed**		
	**β**	**Lower Bound**	**Upper Bound**	**β**	**Lower Bound**	**Upper Bound**	**β**	**Lower Bound**	**Upper Bound**
Coffee ID	0.000	−0.143	0.144	**−0.191**	−0.355	−0.027	−0.155	−0.374	0.063
Roasted	0.023	−0.015	0.061	−0.025	−0.089	0.039	−0.017	−0.087	0.052
Sweet aromatic	0.021	−0.058	0.100	−0.091	−0.237	0.055	−0.071	−0.218	0.076
Nutty	−0.023	−0.069	0.023	−0.048	−0.086	−0.011	−0.043	−0.067	−0.018
Burnt	0.024	−0.015	0.062	0.031	−0.034	0.096	0.028	−0.028	0.085
Woody	−0.010	−0.083	0.062	−0.132	−0.301	0.038	−0.109	−0.220	0.002
Bitter aromatic	0.020	−0.005	0.046	−0.006	−0.101	0.088	−0.002	−0.063	0.059
Cooked	−0.024	−0.061	0.013	−0.005	−0.042	0.033	−0.007	−0.034	0.020
Beany	−0.023	−0.085	0.039	**−0.089**	−0.175	−0.003	**−0.075**	−0.106	−0.044
Dark brown	0.024	−0.025	0.074	**0.059**	0.001	0.117	0.052	−0.001	0.104
Wax	−0.024	−0.062	0.015	−0.031	−0.147	0.084	−0.029	−0.104	0.047
Caramel	0.025	−0.014	0.063	0.023	−0.061	0.107	0.022	−0.041	0.086
Green	−0.015	−0.074	0.044	−0.084	−0.196	0.027	−0.071	−0.163	0.021
Grain	0.025	−0.019	0.069	0.040	−0.022	0.102	0.036	−0.009	0.081
Sesame	−0.006	−0.080	0.069	0.127	−0.046	0.301	**0.103**	0.011	0.194
Sweet taste	0.023	−0.038	0.084	−0.059	−0.198	0.080	−0.045	−0.176	0.086
Bitter taste	0.012	−0.045	0.069	−0.082	−0.183	0.020	−0.065	−0.143	0.013
Balanced/Blended	−0.025	−0.073	0.023	**−0.055**	−0.104	−0.006	**−0.048**	−0.092	−0.005
Fullness	−0.024	−0.091	0.044	**−0.084**	−0.141	−0.026	**−0.072**	−0.117	−0.026
Gel-like	−0.006	−0.125	0.113	**0.169**	0.016	0.322	0.136	−0.019	0.292
Body	−0.024	−0.058	0.010	−0.002	−0.065	0.061	−0.005	−0.047	0.037
Powdery	0.021	−0.063	0.105	**0.123**	0.032	0.213	**0.103**	0.040	0.166
Fat feel	−0.023	−0.061	0.015	−0.020	−0.077	0.036	−0.020	−0.064	0.024
Mouth coating	−0.022	−0.052	0.008	−0.003	−0.060	0.054	−0.006	−0.039	0.027
**Sensory** **Attributes**	**Grouchy**			**Irritated**			**Unfulfilled**		
	**β**	**Lower Bound**	**Upper Bound**	**β**	**Lower Bound**	**Upper Bound**	**β**	**Lower Bound**	**Upper Bound**
Coffee ID	−0.151	−0.314	0.011	−0.081	−0.299	0.137	−0.195	−0.425	0.035
Roasted	−0.016	−0.079	0.047	0.027	−0.027	0.081	−0.022	−0.082	0.038
Sweet aromatic	−0.068	−0.196	0.059	−0.004	−0.157	0.148	−0.090	−0.263	0.084
Nutty	−0.043	−0.106	0.021	**−0.059**	−0.107	−0.010	**−0.053**	−0.083	−0.023
Burnt	0.029	−0.040	0.098	**0.052**	0.024	0.080	0.035	−0.027	0.098
Woody	−0.106	−0.264	0.052	−0.073	−0.209	0.062	−0.136	−0.312	0.039
Bitter aromatic	−0.001	−0.086	0.084	0.031	−0.023	0.085	−0.003	−0.079	0.073
Cooked	−0.008	−0.056	0.040	−0.041	−0.077	−0.005	−0.009	−0.041	0.023
Beany	−0.075	−0.187	0.038	**−0.076**	−0.149	−0.002	**−0.094**	−0.142	−0.047
Dark brown	0.052	−0.031	0.134	**0.065**	0.010	0.120	**0.064**	0.010	0.119
Wax	−0.029	−0.139	0.080	−0.052	−0.110	0.005	−0.036	−0.127	0.055
Caramel	0.023	−0.067	0.113	0.051	−0.024	0.125	0.028	−0.047	0.102
Green	−0.070	−0.198	0.058	−0.061	−0.148	0.026	−0.089	−0.190	0.012
Grain	0.037	−0.047	0.120	0.058	−0.003	0.120	0.045	0.001	0.090
Sesame	0.100	−0.070	0.269	0.045	−0.098	0.188	0.129	−0.026	0.284
Sweet taste	−0.043	−0.159	0.073	0.012	−0.116	0.140	−0.057	−0.209	0.096
Bitter taste	−0.062	−0.172	0.047	−0.015	−0.093	0.063	−0.081	−0.169	0.007
Balanced/Blended	−0.048	−0.130	0.033	**−0.064**	−0.119	−0.010	**−0.061**	−0.099	−0.022
Fullness	−0.071	−0.169	0.027	**−0.075**	−0.151	0.001	**−0.090**	−0.123	−0.056
Gel-like	0.133	−0.037	0.303	0.062	−0.133	0.256	0.171	−0.006	0.349
Body	−0.006	−0.074	0.061	−0.040	−0.098	0.017	−0.006	−0.060	0.048
Powdery	0.101	−0.033	0.235	0.087	−0.024	0.198	**0.129**	0.057	0.200
Fat feel	−0.021	−0.083	0.042	−0.047	−0.093	−0.001	−0.025	−0.076	0.027
Mouth coating	−0.007	−0.064	0.050	−0.038	−0.080	0.004	−0.007	−0.051	0.038
**Sensory** **Attributes**	**Uninspired**								
	**β**	**Lower Bound**	**Upper Bound**						
Coffee ID	−0.169	−0.363	0.024						
Roasted	−0.013	−0.073	0.047						
Sweet aromatic	−0.072	−0.213	0.069						
Nutty	**−0.052**	−0.084	−0.021						
Burnt	0.037	−0.020	0.094						
Woody	−0.121	−0.262	0.020						
Bitter aromatic	0.003	−0.067	0.073						
Cooked	−0.014	−0.053	0.025						
Beany	**−0.088**	−0.125	−0.051						
Dark brown	**0.063**	0.014	0.111						
Wax	−0.038	−0.124	0.049						
Caramel	0.031	−0.035	0.096						
Green	−0.081	−0.164	0.001						
Grain	0.046	0.004	0.087						
Sesame	0.111	−0.005	0.226						
Sweet taste	−0.043	−0.172	0.086						
Bitter taste	−0.068	−0.136	0.001						
Balanced/Blended	**−0.059**	−0.089	−0.030						
Fullness	**−0.084**	−0.116	−0.053						
Gel-like	**0.147**	0.013	0.281						
Body	−0.012	−0.061	0.038						
Powdery	**0.117**	0.079	0.156						
Fat feel	−0.028	−0.080	0.025						
Mouth coating	−0.012	−0.059	0.036						
